# Modes of (Inter)Actions of Polyvalent Immunoglobulins: Nonclinical and Clinical Research in Severe Bacterial Infections

**DOI:** 10.3390/biomedicines14020399

**Published:** 2026-02-09

**Authors:** Sabrina Weißmüller, Carolin Schmidt, Corina C. Heinz

**Affiliations:** 1 Department of Preclinical Research, Biotest AG, 63303 Dreieich, Germany; sabrina.weissmueller@biotest.com; 2 Department of Corporate Project and Portfolio Management, Biotest AG, 63303 Dreieich, Germany; 3 Department of Corporate Clinical Research and Development, Biotest AG, 63303 Dreieich, Germany; carolin.schmidt@grifols.com; 4 Department of Clinical Development, Grifols, 60528 Frankfurt, Germany

**Keywords:** bacterial infection, complement, human polyvalent immunoglobulins, immunomodulation, infectious disease, inflammation, molecular mechanisms, neutrophils, sepsis, therapeutic management

## Abstract

In severe bacterial infections, endotoxin- and exotoxin-induced inflammation and tissue damage, combined with the consequent excessive production of inflammatory mediators by neutrophils, may result in sepsis, septic shock, organ failure, and possibly death. Evidence suggests that supplementation with polyvalent intravenous (IV) immunoglobulin (Ig) preparations, such as standard IVIg or IgM/IgA-enriched Ig preparations, could be an additional treatment option. However, their use in severe bacterial infections like sepsis and septic shock is still a matter of debate. This review summarizes the diverse beneficial mechanisms of (inter)actions of Igs with pathogens and the host. Support for these mechanisms comes from numerous nonclinical studies, complemented by clinical research in adult patients with sepsis, septic shock, and other severe infectious diseases. Depending on Ig type, timepoint of administration, patient population, and dose, the pathogen- and host-induced inflammatory responses are modulated by the combined (inter)actions of polyvalent IgM, IgA, and IgG, with pathogens, and particularly with the host’s neutrophil and complement pathways. However, while nonclinical and clinical studies suggest potential benefits of Ig therapy, clinical evidence remains heterogeneous, and trials with low risk of bias have not consistently demonstrated a definitive survival benefit. A deeper understanding of the conditions under which Ig treatment benefits patients with severe bacterial infections will help select patients most likely to profit from Ig treatment and achieve better outcomes.

## 1. Introduction

Despite the availability of a broad range of antibiotics, morbidity and mortality rates associated with severe bacterial infections remain high [1,2,3]. Inadequate or delayed antimicrobial treatment can significantly increase the risk of progression to life-threatening conditions like sepsis and septic shock.

Sepsis management continues to be a critical challenge in intensive care. To date, only a few adjunctive treatments for sepsis/septic shock have received guideline recommendations [4,5,6]. Especially with the limited therapeutic options available beyond antibiotics and corticosteroids, additional treatments that support pathogen clearance and the host’s immune system may bridge the gap between current strategies and patients’ needs. It is therefore important to deepen the knowledge and understanding of the currently debated therapeutic options to potentially achieve better patient outcomes in the future.

Polyvalent immunoglobulins (Igs) have emerged as a potential adjunctive therapy in severe bacterial infections [7,8]. Details on the various basic characteristics and functions of the different Ig isotypes are summarized in Table 1 [9,10,11,12,13,14,15,16,17] and are further explained in the sections below.

Intravenous immunoglobulin (IVIg) G products such as Flebogamma DIF (Grifols, Barcelona, Spain), Intratect (Biotest, Dreieich, Germany), KIOVIG (Takeda, Vienna, Austria), Privigen (CSL Behring, Marburg, Germany), and Gammagard (Baxter, Deerfield, IL, USA) are widely used for immunodeficiency disorders. These preparations contain more than 95% polyvalent IgG and, according to their package inserts, are not commonly used for acute infections, but are administered to patients at risk of infection [18,19]. Polyvalent Ig preparations enriched with IgM and IgA have been evaluated for use in acute severe infectious diseases [8]. Pentaglobin (Biotest, Dreieich, Germany; 12% IgM, 12% IgA, 76% IgG) is marketed for treatment of severe bacterial infections in several countries. Trimodulin (Biotest, Dreieich, Germany; ~23% IgM, ~21% IgA, ~56% IgG) is currently in clinical development as adjunctive treatment for severe community-acquired pneumonia (sCAP) [20,21].

In bacterial infections, Igs can act through several mechanisms:

1. Binding of endotoxins (i.e., opsonization) and clearance of pathogens;
2. Neutralization of bacterial exotoxins;
3. Modulation of the host inflammatory response.

Understanding the mechanisms of (inter)action of Igs at the experimental level and reviewing available clinical data may help to improve future clinical praxis in the management of sepsis. Therefore, this review summarizes the evidence on the three postulated basic mechanisms of Ig (inter)actions. These specific actions on pathogens and their toxins (Section 2), as well as broader interactions with the host immune system (Section 3), are documented by a wide range of nonclinical studies and supported by numerous clinical investigations conducted in patients with severe bacterial infections. To assemble the evidence for this review, we conducted a comprehensive manual literature search in PubMed, Cochrane Library, and Embase covering publications from January 1985 to December 2025. Clinical studies were included if they investigated the use of polyvalent immunoglobulin preparations (IVIg or IgM/IgA-enriched Ig) in severe bacterial infections, sepsis, or septic shock. Nonclinical studies were considered if they provided mechanistic insights into immunoglobulin–pathogen or immunoglobulin–host interactions. As this is a narrative review, no formal systematic protocol was applied, but emphasis was placed on peer-reviewed studies and meta-analyses most relevant to the scope of this work.

## 2. Activity of Polyvalent Immunoglobulins on the Pathogens

Ig preparations exhibit a range of activities against pathogens, by binding to a large variety of epitopes across different microbial strains. Their opsonizing activity depends on the quantity of specific titers, the manufacturing process [22,23,24,25,26,27], and the regional origin of the plasma used in production [28,29,30]. This variability can even extend to different batches of the same Ig product, and activity varies between different types of Ig preparations [22,31,32,33,34,35,36,37,38].

Commercially available polyvalent IVIg preparations contain mainly IgG (≥95%), which binds to a similar wide range of pathogen epitopes. Preparations that also contain substantial amounts of IgM and IgA may however offer additional benefits by binding to alternative pathogen epitopes, extending pathogen clearance.

### 2.1. Binding of Immunoglobulins to Bacterial Virulence Factors via Opsonization and Clearance of Pathogens

#### 2.1.1. Surface-Bound Bacterial Virulence Factors

Igs neutralize bacterial virulence factors by binding to surface components of pathogens. In Gram-negative bacteria, the membrane-bound endotoxin lipopolysaccharide (LPS), a major virulence factor, can hinder or intensify the functions of polymorphonuclear leukocytes (PMNs) and mononuclear phagocytes (MNs), activate immune responses, induce the secretion of inflammatory cytokines (mainly via Toll-like receptor [TLR] 4 activation) by innate immune cells, cause tissue damage, and mediate endotoxic shock [39]. In Gram-positive bacteria, the most potent virulence factors are major cell wall components such as peptidoglycan and lipoteichoic acid (LTA), which have antigenic properties and stimulate immune cell responses, mainly via TLR2 activation.

Endotoxins are mainly released from bacteria upon lysis, for example, in response to β-lactam antibiotics. Opsonization with Ig inhibits the harmful immune-modulating activities of such virulence factors and enhances their clearance via phagocytosis [29,30,40,41,42].

#### 2.1.2. Nonclinical Studies on Opsonization of Bacterial Virulence Factors and Clearance

Both IVIg and IgM/IgA-enriched Ig preparations induce opsonic killing by PMNs [30]. IVIg induced higher PMN killing rates against *Enterococcus faecium* and *Klebsiella pneumonia*, and IgG was also found to have a higher affinity for the glycerol phosphate backbone of LTA. However, in the presence of complement, the IgM/IgA-enriched Ig induced significantly higher PMN killing rates against multi-resistant nosocomial pathogens like *Escherichia* (E.) *coli*, *Pseudomonas* (P.) *aeruginosa*, and *Staphylococcus* (S.) *aureus* than standard IVIg preparations. Due to its pentameric structure, IgM demonstrates stronger binding affinity for cell wall carbohydrate antigens such as polysaccharides of LPS [30].

These findings were supported by other in vitro studies. Ig levels targeting LPS from pathogen serotypes occurring frequently in Gram-negative septicemia were significantly higher in the IgM/IgA-enriched Ig compared with IVIg. In mouse models infected with *Salmonella typhimurium* or *P. aeruginosa*, survival rates were significantly higher (*p* < 0.05) with IgM/IgA-enriched Ig compared to IVIg [25,37]. IgM antibodies were found to bind LPS, whereas both IgG and IgM antibodies targeted *Yersinia enterocolitica* outer membrane proteins [35,43,44]. Additionally, anti-LPS, anti-flagellum, and anti-outer membrane proteins of *Campylobacter jejuni* were detected in the IgM fraction, indicating species specificity and clearance capacity of the IgM isotype [43,44].

IgA molecules also contribute to neutralizing activities of virulence factors. For example, IgA molecules binding to the M protein of group A streptococci disrupt pathogen adherence to host pharyngeal cells [45]. Similarly, IgA interaction with the O antigen of *Shigella flexneri* suppresses the bacterial type 3 secretion system, which is critical for intestinal epithelial cell invasion [46].

The role of Ig in neutralizing bacterial toxins and enhancing pathogen clearance has also been demonstrated in various sepsis models. In a rabbit model of endotoxin-induced shock, IVIg significantly reduced meningococcal endotoxin levels [47]. IVIg also decreased bacterial organ colonization and increased neutrophil recruitment at the infection site in mouse and rabbit models of streptococcal toxic shock and *E. coli*-induced sepsis [48,49]. Similarly, high-dose IVIg significantly reduced endotoxin levels and improved 7-day survival in a rat model of cecal ligation and puncture (CLP)-induced sepsis [50].

Prophylactic treatment with an IgM/IgA-enriched Ig in a rabbit model of Gram-negative bacteremia improved clearance of *E. coli* from the liver and spleen compared with controls [51]. In another rabbit model of LPS endotoxemia and *E. coli*-induced sepsis, the same preparation accelerated bacterial clearance from the blood. PMNs incubated with the IgM/IgA-enriched Ig exhibited higher phagocytic capacity than the albumin control, as evidenced by increased bacterial uptake without a corresponding rise in oxidative burst [52]. In line with these findings, prophylactic administration of an IgM/IgA-enriched Ig in a murine model of pneumococcal pneumonia (*S. pneumoniae*) significantly reduced bacterial burden in primary (lungs and bronchoalveolar lavage) and secondary target organs (blood and spleen), effectively preventing the development of bacteremia. Histological analyses revealed that, while untreated animals exhibited widespread bacterial dissemination across lung compartments, treated mice had bacteria confined to the alveolar parenchyma, with a progressive reduction in bacterial load over time [53]. This antibacterial effect was exerted through cell-mediated opsonophagocytosis.

In a Gram-positive murine bacteremia model, administration of IgM/IgA-enriched Ig significantly reduced *S. aureus* colonization—4.5-fold in kidneys and 2-fold in liver—compared to IVIg treatment [30]. This higher clearance may be attributed to IgM-mediated complement deposition and effective complement-dependent opsonophagocytosis (see Section 3).

#### 2.1.3. Clinical Studies on Endotoxin Opsonization and Bacterial Clearance

Prospective clinical studies investigated the anti-endotoxin activity of IgM/IgA-enriched Ig preparations in patients with signs of Gram-negative sepsis or septic shock. In a pivotal study on Gram-negative sepsis, serum endotoxin concentrations decreased during the first 24 h of treatment and were significantly lower in survivors compared to non-survivors (*p* < 0.01). Mortality was also significantly reduced in the treatment group (4%) vs. the control group (32%, *p* < 0.01) [54].

In a cohort of patients with cancer and suspected Gram-negative sepsis, IgM/IgA-enriched Ig significantly reduced endotoxin plasma levels within the first 18 h (*p* < 0.05). Additionally, serum levels of IgM and IgG antibodies against lipid A and LPS were significantly elevated (*p* < 0.01, repeated measure ANOVA) [55]. A smaller study on patients with severe sepsis found significant reductions in endotoxin levels within 6 h (*p* = 0.01) and 12 h (*p* = 0.003) post-treatment compared to controls [56].

Further supportive evidence comes from a randomized, double-blind, placebo-controlled clinical trial in patients with Gram-negative bacteremia. Administration of a monoclonal IgM anti-endotoxin antibody targeting the lipid A domain significantly reduced mortality (30%) compared to controls (49%, *p* = 0.014) [57]. Further details and additional clinical studies on endotoxin activity are summarized in Supplementary Table S1 [54,55,56,57,58,59,60,61,62,63,64,65,66,67,68,69,70,71,72].

Despite these encouraging findings with monoclonals, a meta-analysis [73] of various randomized controlled trials (RCTs) in patients with severe sepsis or septic shock found no overall survival benefit from monoclonal IgM anti-endotoxin antibodies (anti-E5 and HA-1A) (risk ratio [RR] = 1.01; 95% confidence interval [CI]: 0.94–1.09; 8 trials; n = 4443) [57,58,74,75,76,77,78,79], while anti-cytokines demonstrated a marginal reduction in mortality (RR = 0.92; 95% CI: 0.86 to 0.97; 9 trials; n = 7893). In contrast, subgroup analysis in the same Cochrane meta-analysis identified a significant reduction in mortality among adults with sepsis treated with IVIg preparations (RR = 0.81; 95% CI: 0.70–0.93; 10 trials; n = 1430) or IgM/IgA-enriched Ig preparations (RR = 0.66; 95% CI: 0.51–0.85; 7 trials; n = 528) [73]. However, a sensitivity analysis of trials with low risk of bias did not show a reduction in mortality in adults. Hence, caution must be taken in the interpretation of statistically significant results from the pooled analysis of small studies [73].

Many of the available studies were conducted decades ago, before the implementation of standardized sepsis protocols, and often involved small sample sizes, inconsistent timing of Ig administration, or insufficient stratification by disease severity. Even so, their findings highlight the importance of polyvalent Ig in providing broader immune support compared to monoclonal approaches, as they engage multiple mechanisms of pathogen clearance beyond endotoxin neutralization.

### 2.2. Neutralization of Exotoxins by Polyvalent Immunoglobulins

#### 2.2.1. Secreted Bacterial Virulence Factors (Exotoxins)

In the management of severe bacterial infections, neutralization of bacterial exotoxins plays a crucial role, particularly when dealing with pathogens that employ exotoxins as virulence factors. Unlike antibiotics, which primarily target bacterial growth, polyvalent Igs can directly bind and neutralize these exotoxins.

Exotoxins are highly diverse, and their classification can be complex. Classification based on mechanism of action differentiates superantigens, membrane-disrupting toxins, and A-B toxins.

Superantigens bypass normal antigen presentation and cause excessive immune responses by manipulating immune cell functions, for example by binding to T cells and APCs via the major histocompatibility complex II and the T-cell receptor. This interaction may induce T-cell proliferation and massive cytokine release, leading to inflammation. Examples of superantigens are streptococcal pyrogenic toxin superantigens (PTSAgs), streptococcal pyrogenic exotoxin A (SpeA, from *S. pyogenes* causing streptococcal toxic shock syndrome [STSS]), erythrogenic toxins, and staphylococcal PTSAgs, including staphylococcal enterotoxins (e.g., SE-A, SE-B) and toxic shock syndrome toxin-1 (TSST-1) [80,81,82,83].

Membrane-disrupting or pore-forming exotoxins include alpha-hemolysin (from *Staphylococcus aureus*), Streptolysin O (from *Streptococcus pyogenes*), and alpha-toxin (from *Clostridium*), which can damage cell membranes by forming pores or hydrolyzing phospholipids, resulting in cell lysis (like red blood cells and platelets) [84,85].

A-B toxins like *Diphtheria* and *Cholera* toxins and exotoxin A (from *Pseudomonas*) have an enzymatic (A) component and a binding (B) component that allows attachment to host cells. They enter the cell via endocytosis and after endosomal proteolytic cleavage, the A subunit acts enzymatically on host proteins [86].

#### 2.2.2. Nonclinical Studies on Neutralization of Exotoxins by Immunoglobulin Preparations

In vitro studies have consistently shown the efficacy of Ig preparations in neutralizing certain bacterial exotoxins, particularly those produced by *S. aureus* and *S. pyogenes*, as shown in Table 2 [33,35,49,87,88,89,90,91,92,93,94,95,96,97,98,99,100,101]. The strong neutralizing effects observed in vitro are corroborated by numerous in vivo studies examining poly-specific Igs, which show similar results in reducing toxin effects [25,33,49,93,94]. These findings highlight the clinical potential of Ig therapies in managing toxin-induced pathologies, especially in intensive care settings where rapid toxin neutralization can be crucial. Nevertheless, it has to be noted that differences in the exotoxin-neutralizing activity of different exotoxins by different types of Ig preparations have been shown. Furthermore, these observations are derived from experimental settings. Comparative clinical evidence confirming superior toxin-neutralizing activity of one Ig preparation over another in a specific toxin-related indication remains limited.

#### 2.2.3. Clinical Studies on Exotoxin Neutralization

The relationship between low anti-exotoxin antibody levels at infection onset and poor outcomes in invasive streptococcal disease is well documented [102,103,104,105,106]. In patients with bacteremia and invasive *S. pyogenes* infections, insufficient antibody levels have been linked to increased mortality [104,107,108,109,110].

Beyond the studies summarized in Supplementary Table S1 [54,55,56,57,58,59,60,61,62,63,64,65,66,67,68,69,70,71,72], several additional clinical investigations have explored the efficacy of Ig preparations in providing anti-exotoxin antibodies to patients with severe bacterial infections. One retrospective study reported that IVIg treatment enhanced serum neutralizing activity against *Clostridium difficile* toxins A and B in patients with recurrent infection, with partial therapeutic response observed in approximately 41% of cases. However, variability in efficacy was noted, likely due to differences in IVIg preparations and baseline antibody levels [111].

In the context of streptococcal toxic shock syndrome (STSS), a meta-analysis of small studies showed reduced mortality (from 33.7% to 15.7%) in patients treated with clindamycin and IVIg [112]. STSS, caused by invasive group A *Streptococcus*, involves superantigens and other virulence factors that trigger septic shock and multiorgan failure, with mortality rates reaching up to 50%. Although the meta-analysis suggested potential benefits of IVIg in STSS, the evidence of the small studies remains weak. Due to the rarity of the STSS condition, clinical evaluation remains challenging; one randomized controlled trial was prematurely terminated because of slow recruitment [62]. The necessity for the use of placebo-controlled designs further complicates robust evaluation in this rare disease. Nevertheless, a more recent broader review of the clinical and nonclinical literature further supports the use of clindamycin and IVIg as adjunctive therapies in managing invasive group A *Streptococcus* infections, when possible, to reduce mortality risk [113].

Given these insights, a more targeted approach that combines toxin neutralization with antimicrobial therapy may improve outcomes in critically ill patients with severe bacterial infections. Nonetheless, further clinical trials with larger sample sizes are needed to confirm the role of polyvalent Igs in this context. To overcome the hurdles in rare diseases, future clinical research in such indications could be focused on adaptive trial designs using Bayesian statistics and based on the effect size observed in the meta-analysis. This would likely require fewer patients than classical RCTs and would allow early stopping for benefit. The collection of data in a prospective, international disease registry with embedded comparative effectiveness could, for example, be another option.

### 2.3. The Additive Effect of Polyvalent Immunoglobulins and Antibiotics

#### 2.3.1. Antibiotics Limitations and Additive Effect of Polyvalent Immunoglobulins

Antibiotics exert their therapeutic effects by targeting specific bacterial processes such as cell wall biosynthesis, protein synthesis, nucleic acid metabolism, and cellular integrity [114]. For example, β-lactams inhibit peptidoglycan synthesis, fluoroquinolones disrupt DNA replication, and glycopeptides block cell wall construction. However, antibiotics alone are not always sufficient in managing severe bacterial infections. Several limitations affect their efficacy, including uncertainty about the causative pathogen at presentation, the release of pathogen-associated molecular patterns (PAMPs) that exacerbate inflammation, and the growing prevalence of multidrug-resistant (MDR) bacteria.

**Figure 1:**
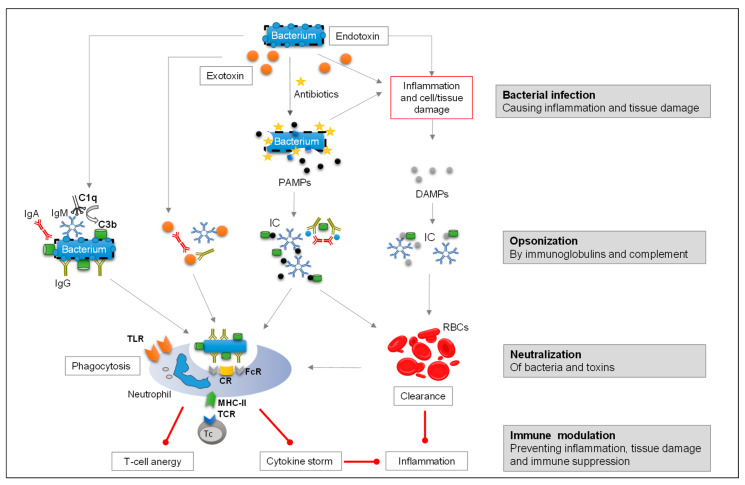
Modes of action of Igs on the pathogen and interactions with complement and neutrophils. Bacteria cause systemic inflammation and tissue damage (release of DAMPs, gray circles) via endotoxins (blue circles) present in their membrane or via secreted exotoxins (orange circles). Additionally, bacterial debris (PAMPs, black circles and dashed black lines) generated by antibiotic treatment (yellow asterisks) may cause inflammation and tissue damage if clearance is not sufficient. Jointly, toxins, PAMPs, DAMPs, and cytokines stimulate neutrophils. A cytokine storm may develop, and immune-mediated tissue damage further fuels persistent inflammation. In addition to cytokines, neutrophils secrete reactive oxygen species and proteases. Persistent pro- and anti-inflammatory responses and overstimulation (proliferation) of T cells may lead to pathological T-cell responses (e.g., apoptotic depletion, decreased proliferation, and T helper 2 cell polarization and unresponsiveness [T-cell anergy]). In the presence of sufficient amounts of Igs, bacteria and their toxins, dead bacteria, and PAMPs are opsonized with antibodies. In the presence of IgM and IgG, additional complement opsonization facilitates fast clearance of bacteria, PAMPs, and DAMPs via local phagocytosis by neutrophils or via transport on the RBCs to phagocytes in the liver. This prevents overstimulation of neutrophils, persistent inflammation, tissue damage, and immune suppression (shown with red circle-headed lines). C1q: complement factor C1q; C3b: complement factor C3b (green box); CR: complement receptor (yellow box); DAMPs: damage-associated molecular patterns; FcR: Fc receptor (gray arrow); ICs: immune complexes; Ig: immunoglobulin; MHC-II: major histocompatibility complex II (green arrow); PAMPs: pathogen-associated molecular patterns; RBCs: red blood cells; Tc: cytotoxic T cell; TCR: T-cell receptor (blue arrow); TLR: Toll-like receptor (orange arrow).

Polyvalent Ig preparations can mitigate some of these challenges. Their broad-range activity makes them particularly useful in severe infections where the pathogen is unidentified. In addition, Igs can limit tissue damage by aiding the opsonization and subsequent phagocytosis of PAMPs and pathogens, including MDR strains (Figure 1) [115].

#### 2.3.2. Nonclinical Studies on the Additive Effects of Immunoglobulins and Antibiotics

The additive effect of polyvalent Ig preparations combined with antibiotics has been investigated in in vitro settings, confirming the additive effect of Ig against MDR bacteria including methicillin-resistant *S aureus* (MRSA) [116]. This is supported by different animal models of infections [93,94]. In a rat model of abdominal infection, treatment with IgM/IgA-enriched Ig significantly reduced *E. coli* bacterial load in the blood when administered alongside imipenem, suggesting enhanced bacterial clearance compared to the control group treated with imipenem plus albumin [117].

In mouse models, the combination of IgM/IgA-enriched Ig with either ampicillin or cefsulodin immediately after intraperitoneal infection with *S. aureus* or *P. aeruginosa* showed additive effects on survival rates (*S. aureus*: 90% of animals survived with combination therapy vs. 60% with antibiotics alone; *P. aeruginosa*: 70% with combination therapy vs. 20% with antibiotics alone) [25].

Further evidence comes from a study on a murine model, where treatment with IVIg three hours after intranasal infection with *S. pneumoniae* resulted in effective bacterial clearance from blood and lungs after 48 h. Survival rates were markedly higher in the group receiving both IVIg and ampicillin compared to either treatment alone [118]. Additional murine models have also demonstrated enhanced protective effects when IVIg preparations were combined with antibiotics [119,120].

Consistent with these findings, the additive effect of ampicillin and IgM/IgA-enriched Ig was also demonstrated in vitro: while ampicillin inhibited *S. pneumoniae* growth, the Ig preparation, in the presence of neutrophil-like granulocytes and complement, reduced colony-forming units via opsonophagocytosis. In a complex murine model of pneumococcal pneumonia followed by mechanical ventilation, the combination of both treatments enhanced bacterial clearance and significantly reduced systemic inflammatory protein levels in ventilated animals, suggesting additive antibacterial and anti-inflammatory effects [53].

A more indirect additive effect has been suggested specifically for IgA. Extended use of broad-spectrum antibiotics can deplete the gut microbiota, impairing the production of protective secretory IgA by resident plasma B cells. In mice, treatment with purified *P. aeruginosa*-specific IgA following antimicrobial therapy enhanced resistance to this nosocomial pathogen [121].

Overall, these studies emphasize the potential of Ig not only to neutralize pathogens directly, but also to improve the efficacy of antibiotics, particularly in the context of MDR pathogens.

#### 2.3.3. Clinical Studies on the Additive Effects of Polyvalent Immunoglobulins and Antibiotics

Although additive effects are consistently observed in animal models, clinical confirmation remains incomplete, and translation of such preclinical combinations to patient benefit is ongoing. Recent clinical research has highlighted the potential adjunctive benefits of Ig therapy in the treatment of sepsis and septic shock, particularly in patients infected with MDR pathogens such as extended-spectrum β-lactamase-producing *E. coli* and *P. aeruginosa*, where standard treatments often face limitations (Supplementary Table S1 [54,55,56,57,58,59,60,61,62,63,64,65,66,67,68,69,70,71,72]). Whereas IVIg preparations are routinely used as prophylaxis in immunocompromised patients, IgM/IgA-enriched Ig preparations have been administered in combination with antibiotics to improve recovery in patients staying in the intensive care unit (ICU) with MDR bacterial infections [122].

Additional clinical studies have investigated this combined approach. In a case series involving six immunocompromised hematopoietic stem cell transplant patients, IVIg enhanced neutrophil-mediated killing of MDR *P. aeruginosa* and extended-spectrum β-lactamase-producing *E. coli* by increasing superoxide release and autophagy [123]. Another report described two patients with hypogammaglobulinemia and persistent *Campylobacter jejuni* infections who did not respond to a combination of antibiotics and IgG, but achieved pathogen clearance following treatment with IgM/IgA-enriched Ig [59]. However, even if these case studies are supported by in vitro data discussed above (Section 2.3.2), the comparative evidence from clinical data between IVIg and IgM/IgA remains too limited for definitive conclusions on an Ig-type-related treatment benefit in this indication.

A retrospective study of 94 patients with sepsis or septic shock due to MDR bacteria found that, among various adjunctive interventions, only the administration of IgM/IgA-enriched Ig resulted in statistically significant improvement in survival during ICU stay (*p* = 0.011) [60]. Similarly, another retrospective study on patients with hospital-acquired severe infections due to MDR Gram-negative bacteria showed that early administration (within 24 h of infection onset) of IgM/IgA-enriched Ig, in addition to appropriate antibiotics, significantly reduced the 28-day all-cause mortality (39% with combination therapy vs. 58% with antibiotics alone, *p* = 0.011) and extended protection from breakthrough bacteremia (7–12 days vs. 3–6 days, *p* < 0.0001). In a subgroup of patients infected with extremely drug-resistant (XDR) Gram-negative bacteria, mortality was also significantly lower in the Ig-treated group (38.5% vs. 62.9%, *p* = 0.008) [66].

Even though case studies and retrospective studies indicate improved survival when IgM/IgA-enriched Ig is combined with antibiotics in MDR infections, the current evidence is still limited and requires results from prospective, randomized, and/or comparative trials conducted with a low risk of bias.

### 2.4. Summary of Section 2

Ig preparations have various antibacterial activities that are additive to those of antibiotics. The inclusion of IgM and IgA in IgG preparations might broaden the overall protective activities of Ig preparations. Nevertheless, most comparative data arises from preclinical studies, and definitive clinical evidence demonstrating superior effectiveness of IgM/IgA-enriched preparations over standard IVIg remains limited. Based on the available nonclinical evidence, IgG may be in some cases more effective in exotoxin neutralization, whereas IgM may add important anti-endotoxin activity. Moreover, IgM seems to play a more prominent role in the clearance of pathogens that secrete exotoxins. Observed beneficial clinical effects of Ig preparations include toxin neutralization and reduction in mortality from sepsis and septic shock as well as additive survival effects in critically ill patients facing infections from MDR/XDR pathogens. This adjuvant, broad-repertoire treatment strategy might be preferable in cases where the causative pathogen is unknown or shows antimicrobial resistance.

## 3. Activity of Polyvalent Immunoglobulins on the Host

### 3.1. Modulation of Neutrophil (Opsono)Phagocytosis Functions by Immunoglobulins

#### 3.1.1. Phagocytosis by Neutrophils

Neutrophils play a central role in the immune defense against bacterial infections, particularly through phagocytosis. Antibodies bind pathogens using their Fab regions, while their Fc regions engage Fc receptors (FcRs) on neutrophils and other phagocytes, thus facilitating the clearance of immune complexes (Figure 1). This process is essential to rapidly eliminate pathogens, especially in patients facing sepsis or systemic inflammatory responses. Key phagocytes include many types of PMNs like neutrophils and other granulocytes, along with MNs such as monocytes, macrophages, and dendritic cells.

Neutrophils represent the most abundant type of leukocytes in the blood and accumulate at the site of infection and inflammation in the acute stage of the disease. They also reside in marginated pools in lung tissue. In pulmonary infections, these marginated lung-resident neutrophils rapidly recognize and engulf disseminating pathogens, highlighting the importance of lung neutrophil pools in systemic infections [124].

#### 3.1.2. Interactions Between Immunoglobulins and Neutrophil Receptors

Neutrophils express various FcRs that enable binding to different antibody isotypes, as shown in Table 1 [9,10,11,12,13,14,15,16,17].

Quiescent phagocytic cells predominantly express the IgG receptors FcγRII (also known as cluster of differentiation [CD] 32) and FcγRIII (CD16), along with the IgA receptor FcαR (CD89). Upon stimulation by bacterial membrane proteins like LPS and LTA, neutrophils upregulate FcγRI (CD64), a high-affinity IgG1 and IgG3 receptor [125,126]. This upregulation likely occurs through binding of LPS/LTA to TLR4, TLR2, and the neutrophil LPS co-receptor CD14 [127,128], triggering the release of inflammatory mediators and initiating antimicrobial responses. The relevance of IgG-opsonized pathogen phagocytosis is supported by the increased infection susceptibility observed in patients with inheritable defects in Ig production.

During inflammation, neutrophils also increase the expression of IgA receptors (CD89), which enhances the phagocytosis of IgA-opsonized particles [129,130]. Pathogen clearance depends on the polymeric form of IgA and the presence of complement factors C3b and C4b [131]. These so-called opsonins interact with complement receptor (CR)1 and CR3 on MNs and PMNs, promoting opsonophagocytosis—a more effective clearance process than phagocytosis alone [132]. Therefore, both FcαR and CR1/CR3 (i.e., CD35/CD11b) are involved in IgA-mediated phagocytosis. Importantly, polymeric IgA is present in IgM/IgA-enriched Ig preparations [133].

Phagocytic activity is particularly enhanced when pathogens are opsonized by pentameric IgM, which strongly induces complement factor C3b deposition on the pathogen and enables effective opsonophagocytosis via CRs. A specific neutrophil receptor for IgM is likely not expressed.

Interestingly, IgM/IgA-enriched Ig preparations, unlike IVIg preparations, counterbalanced the LPS-induced upregulation of FcγR expression [128,134]. This suggests that higher concentrations of IgM/IgA potentially prevent LPS-induced hyperinflammation and better maintain immune homeostasis. However, this effect has been demonstrated ex vivo, and its magnitude and clinical relevance in patients with sepsis remains to be clarified.

#### 3.1.3. Clinical Studies on Phagocyte Functions in Severe Infections

While a multitude of nonclinical studies robustly support the role of Ig in enhancing phagocytosis of Ig-opsonized particles, direct clinical evidence demonstrating increased phagocytic responses in sepsis patients following Ig treatment remains limited. Indirect proof for this mechanism comes from the expression dynamics of CD64 on phagocytic cells.

Prospective interventional and noninterventional studies showed that CD64 expression transiently increases during the acute inflammatory phase, both in experimental human endotoxemia and in sepsis [135,136,137]. In patients with severe sepsis or septic shock, elevated CD64 expression on PMNs and MNs was favorably correlated with survival, suggesting a link between phagocytic function and clinical outcomes. Notably, reduced phagocytic activity of peripheral blood PMNs at admission was associated with mortality in these patients [138].

In critically ill patients, particularly those with sepsis, the decline in neutrophil CD64 expression signals progression to an immunosuppressed or “hyporeactive” phase due to immune exhaustion. This might be caused by continuous stimulation by bacterial toxins and/or systemic cytokines [139]. The lack of response of neutrophils has also been observed in patients with acute respiratory distress syndrome and severe nosocomial pneumonia [140]. At this stage, IVIg infusions neither changed the expression of FcRs and CRs on phagocytes nor improved their phagocytic activity.

Beyond impaired phagocytosis, neutrophils in sepsis exhibit additional dysfunctions, including delayed apoptosis, impaired recruitment, migration and adhesion, and altered neutrophil extracellular trap (NET) formation, or NETosis [141,142,143,144,145,146,147,148,149,150,151]. Increased NETosis further complicates immune response by promoting inflammatory cascades and exacerbating vascular occlusion, which can impair tissue perfusion and amplify sepsis progression (see Section 3.3.2).

Despite these impairments, Ig therapy may help preserve neutrophil function, as discussed in Section 2. By inducing phagocytosis via Fc receptor binding and neutralizing bacterial toxins, timely administration of Ig could prevent progression to advanced disease stages. Reversing neutrophil functions may promote bacterial clearance via Ig-mediated phagocytosis and improve sepsis outcomes [152,153]. Further clinical studies are needed to confirm this therapeutic potential.

### 3.2. Modulation of Pro- and Anti-Inflammatory Responses by Polyvalent Immunoglobulins

#### 3.2.1. Infection-Induced Inflammatory Responses

During severe bacterial infections, PAMPs, such as endotoxins and exotoxins, along with damage-associated molecular patterns (DAMPs), initiate complex inflammatory immune responses [154,155,156]. PAMPs interact with pattern recognition receptors like TLRs, stimulating phagocytic cells and leading to a cascade of immune events [157]:

1. Neutrophils, monocytes, and macrophages release large amounts of pro-inflammatory cytokines and chemokines (e.g., tumor necrosis factor-alpha [TNF-α], interleukin [IL]-1, IL-6, IL-8, monocyte chemoattractant protein-1 [MCP-1], macrophage inflammatory protein [MIP]1β, interferon [IFN]-γ) at the site of infection (Figure 1).
2. This cytokine storm recruits additional neutrophils by the secretion of chemokines (e.g., CXCL-1, CXCL-2, CXCL-5, IL-8/CXCL-8) by macrophages and injured epithelial cells, thus increasing local phagocytic activity.
3. Excessive local inflammation and tissue damage can provoke systemic responses, which are countered by anti-inflammatory mediators (e.g., IL-4, IL-10, IL-13, and transforming growth factor β) and cytokine inhibitors like soluble TNF receptor 2 and soluble IL-6 receptor, which may be released to avoid widespread damage [158,159,160,161].

Unbalanced and persistent systemic pro-inflammatory and compensatory anti-inflammatory responses (known as systemic inflammatory response syndrome) can ultimately lead to immune suppression, particularly affecting adaptive immunity. This includes decreased antigen presentation, macrophage paralysis, depressed T-cell proliferation and responsiveness (e.g., anergy; Figure 1), increased lymphocyte and dendritic cell apoptosis, and a shift from T helper cell type 1 to T helper cell type 2 phenotype [162,163,164,165,166,167]. Such dysregulation, both locally and systemically, is a crucial factor in sepsis pathogenesis. Timely opsonization and phagocytosis of bacterial endotoxins and other PAMPs, along with exotoxin neutralization, are important to prevent overstimulation of TLR and immune imbalance.

#### 3.2.2. Mechanism of Immune Modulation by Immunoglobulin Preparations

Different mechanisms of Ig-mediated modulation of inflammatory responses have been proposed [168,169,170]:

1. Indirect Ig interference with the TLR/nuclear factor kappa-light-chain-enhancer of activated B cell (NFκB) signaling pathway by binding endotoxins and exotoxins, thereby preventing receptor activation on neutrophils and reducing cytokine expression (Figure 1) [171].
2. Indirect interference with the TLR–MyD88 pathway and direct interaction of IgG with FcγR–ITAM–Syk signaling, which may regulate cytokine production [128,134,172,173].
3. Direct binding of high-avidity antibodies in polyvalent IgG to cytokines like IFN-α, IL-1α, and IL-6 [174,175]. High-dose IVIg therapy (1000–3000 mg/kg) may modulate cytokine activity and exert immunomodulatory and anti-inflammatory effects [176]. Interestingly, IgM/IgA-enriched Ig bind to IL-8 more efficiently at lower concentrations than IVIg [134], suggesting that lower doses may be sufficient for similar effects.
4. Increased release of soluble cytokine receptors that can bind cytokines and prevent their inflammatory actions after IVIg treatment [177].
5. Upregulation of inhibitory FcγRIIB on macrophages after treatment with high-dose IVIg, which was shown to exert an anti-inflammatory effect [175].
6. Increased autophagy of inflammatory cells such as monocytes, dendritic cells, and M1 macrophages [178]. IVIg was found to be able to suppress the inflammatory cytokines in innate immune cells.

These effects may support immune homeostasis and represent an additional therapeutic mechanism for autoimmune and inflammatory diseases.

#### 3.2.3. Nonclinical Studies on Modulation of Inflammatory Responses by Immunoglobulin Preparations

Numerous in vitro studies have shown the cytokine-modulating effects of IVIg preparations. For example, a specific immunomodulatory effect on IL-6 secreted by monocytes and T cells, suppression of pro-inflammatory cytokines such as TNF-α and IL-6 in LPS-stimulated peripheral blood MNs, and upregulating effect on chemokines [171,179,180]. IgM/IgA-enriched Ig preparations have shown similar effects, with reduced secretion of pro-inflammatory cytokines in ex vivo LPS-stimulated monocytes [128,181].

Experimental IgA preparations (containing 95% IgA) have also demonstrated reduced secretion of pro-inflammatory cytokines (IL-6 and TNF-α), anti-inflammatory cytokines (IL-10, IL-12p40), and chemokines (MIP1α, MIP1β, MCP1) after LPS stimulation of peripheral blood MNs or monocytes [182,183,184]. Furthermore, LPS-induced inflammation in neutrophil-like human leukemia (HL)-60 cells was counteracted by the IgA component of IgM/IgA-enriched Ig preparations via FcαRI-dependent inhibitory signaling [134,185]. Thus, the dampening reactivity of IgM/IgA-enriched Ig preparations on cytokine secretion might be in part due to the higher amounts of IgA (12–21%) compared to IVIg preparations (Table 1). However, data on these immunomodulatory differences between IVIg and IgM/IgA-enriched Ig detected in vitro is still limited, requiring further comparative studies that also investigate comparable dose regimens and unravel the functions of the single and combined Ig types.

These in vitro results are supported by in vivo studies. IVIg pre-treatment reduced both IL-6 and TNF-α plasma levels in LPS-infused rats and in murine CLP models, in a dose- and time-dependent manner [186,187]. It also suppressed alveolar epithelial cell apoptosis and reduced acute lung injury [187]. In *P. aeruginosa*-infected mice, early IVIg treatment (three hours after infection) significantly reduced IL-6, IL-1β, and TNF-α levels [188]. In a separate study involving *S. pyogenes*-infected mice, early IVIg administration at the time of infection reduced IL-6 levels; however, this effect was not observed when IVIg was given after the infection was fully established [49].

IgM/IgA-enriched preparations also reduced IL-1β plasma levels and histological injury scores in lung and small-intestine tissues in rat CLP models [189]. Although this study included histopathological scoring and evaluation of polymorphonuclear leukocyte infiltration in lung and intestinal tissues, the authors noted limitations such as the absence of measurements of in situ cytokine release, apoptotic index, and animal survival. More comprehensive experimental studies are therefore needed to confirm and expand these findings.

Regarding the effect of IgM/IgA-enriched Ig on anti-inflammatory IL-10 responses, differences have been noted. In vitro, IgM/IgA-enriched Ig (and IgA preparations) inhibited IL-10 secretion in LPS-stimulated human monocytes [128,182]. Furthermore, it attenuated IL-1β production and increased IL-10 secretion in a porcine sepsis model, and increased serum IL-10 levels in ventilated and LPS-treated rats [189,190,191]. In septic shock patients, IgM/IgA formulations reduced IL-10 serum levels [192], contrasting with animal model findings. These differences may reflect species-specific responses, differences in immune status and inflammatory stage of sepsis, and/or variability in experimental conditions and dosing.

#### 3.2.4. Clinical Studies on Modulation of the Inflammatory Response

Clinical studies investigating the effects of polyvalent Igs on cytokine levels in patients with sepsis are limited by several factors. Cytokine regulation may occur locally in tissues and not be reflected in plasma [193]. Moreover, cytokines have short half-life compared to inflammatory markers such as C-reactive protein (CRP), procalcitonin (PCT), or ferritin, and baseline plasma levels are highly variable among patients. Therefore, the regulation of inflammatory responses has not been used as a primary outcome in clinical studies with Ig, and correlations with clinical outcomes have not been well described so far.

Despite these challenges, several retrospective and prospective studies have been conducted as summarized below and in Supplementary Table S2 [15,69,181,192,194,195,196,197,198,199,200,201,202,203,204,205,206,207,208,209,210].

##### Modulation of Cytokine Levels by Immunoglobulin Preparations

Several studies reported decreased TNF-α and IL-6 levels after treatment with IVIg or IgM/IgA-enriched preparations compared to controls [69,195,196,197,198,211]. This is likely achieved through various Ig-mediated immune modulation mechanisms, as discussed above, including toxin neutralization.

A prospective study on patients with autoimmune diseases confirmed the direct cytokine-neutralizing activity of high-dose IVIg therapy (15 treatment series of 0.4 g IgG/kg/day for 3 days), showing increased cytokine binding to IgG in sera, most likely due to specific anti-cytokine antibodies against IFN-α, IL-1α, and IL-6 [204]. The presence of neutralizing antibodies in IVIg preparations may explain, at least in part, the immunosuppressive and anti-inflammatory effects of high-dose IVIg therapy. This is not only important in autoimmune diseases but also in cases of overshooting inflammatory responses caused by severe infections.

An indirect effect of cytokine modulation is fever reduction. LPS is an exogenous pyrogen that induces leukocytes to release endogenous pyrogens—such as IL-1, IL-6, IFN-γ, and TNF-α—which stimulate prostaglandin E2 production by endothelial cells and initiate fever [212,213,214]. Several prospective studies have shown that IVIg or IgM/IgA-enriched preparations reduce fever faster compared with control groups [63,199,203,215].

##### Modulation of CRP and PCT Levels by Immunoglobulin Preparations

Two prospective studies found that re-increased (after 1 week) or permanently elevated PCT levels were associated with higher mortality rates [216,217]. In contrast, patients treated with IgM/IgA-enriched Ig preparations showed a permanent decrease in PCT and improved outcomes in sCAP and sepsis, including reduced mortality [181,202,203,206,209].

In other clinical studies, PCT and/or CRP levels decreased significantly faster or more steadily and faster in patients treated with IgM/IgA-enriched Ig [181,203,205,206,209] or IVIg [197] compared to the placebo.

A relation between high PCT values and an anti-inflammatory response characterized by human leucocyte antigen-DR isotype (HLA-DR) depression on monocytes was also found [216,218]. A decrease in HLA-DR expression on monocytes is a marker of immune suppression and poor prognosis [219]. Treatment with Ig preparations may accelerate HLA-DR recovery by reducing infection load and rapidly and permanently lowering PCT levels. The effect of IgM/IgA-enriched Ig in patients with peritonitis and low HLA-DR status is currently being investigated [220].

### 3.3. Modulation of Complement Functions by Polyvalent Immunoglobulins

The complement system is an essential defense mechanism against invading pathogens and is composed of a complex network of more than 30 proteins. Activation can occur via three different pathways: classical, alternative, and mannose-binding lectin (MBL). The different Ig isotypes provide different activities within the complement pathways (Table 1) [9,10,11,12,13,14,15,16,17]. The classical pathway is activated by pathogens bound by IgG1, IgG2, IgG3, or IgM through binding to complement factor C1q. The alternative pathway is activated by spontaneous hydrolysis (tick over) of C3. The MBL pathway is activated by recognition of microbial cell surface carbohydrates by MBL or ficolin. The alternative and MBL pathways are also activated by polymeric IgA [221,222].

Deposition of complement factors C3b and C4b on pathogens is induced by activation of one of the three pathways. This process is most efficient via the classical pathway through binding IgM and, to a lesser extent, IgG to pathogens. An in vitro study demonstrated dose-dependent C3b deposition on *E. coli* LPS (O111:B4) by LPS-reactive monoclonal IgM and IgG antibodies [223]. These immune complexes promote opsonophagocytosis by neutrophils via CR and FcR (see Section 3.1) [224]. However, opsonophagocytic activity and efficiency of bacterial killing vary between antibody isotypes (IgM > IgG2a > other IgG subclasses), consistent with their C3-fixing capacity [223,225].

In severe infections such as sepsis, dysregulation of the complement system contributes to hyperinflammation [226]. Due to a shortage of neutrophils or Igs, the terminal complement cascade may be further activated, leading to membrane attack complex (MAC) formation and resulting in pore formation on the pathogen. This drives direct lysis of the pathogen through complement-dependent cytotoxicity (CDC) [227,228]. If CDC occurs excessively, large amounts of bacterial debris, including endotoxins and PAMPs, are released and may cause inflammation and harm the host. The terminal pathway also triggers the release of complement factors C3a and C5a, which are anaphylatoxins that may induce additional inflammatory processes that require control mechanisms (see Section 3.3.2).

#### 3.3.1. Modulation of C3b/C4b Opsonins and Prevention of Excessive Cell Lysis

Polyvalent IgM, and to a lesser extent IgG, appears to have dual concentration-dependent functions in the classical pathway. On one hand, antibodies activate the pathway through binding to the pathogen in a concentration-dependent manner, resulting in the concomitant deposition of the opsonins C3b and C4b on its surface and increasing opsonophagocytic activity. In vitro studies have shown that human IgM and IgA have a higher capacity to bind soluble C3b and C4b than IgG [229]. Furthermore, IgM preparations interact more strongly with complement components (C1q, C3, and C4) than IgG preparations [230,231]. On the other hand, high IgM concentrations (and IgG to a lesser extent) inhibit the complement pathway by scavenging C3b and C4b, thus preventing the terminal cytolytic complement pathway, MAC formation, and CDC (purified IgM > IgM-enriched > IVIg) [15,231,232,233,234,235,236,237,238,239,240]. This activity attenuates excessive anaphylatoxin generation.

Complement binding by polyvalent IgM was confirmed in vivo in a rat anti-Thy 1 nephritis model, where a single dose of IgM preparation prevented glomerular deposition of C3-, C6-, and C5b-9, whereas the effect of IVIg was much less pronounced. Importantly, neither IgM preparations nor IVIg negatively affected the in vitro phagocytosis of *E. coli* by human granulocytes.

#### 3.3.2. Modulation of C3a/C5a Anaphylatoxins and Prevention of Inflammation and NETosis

Anaphylatoxins have various effector functions, including histamine release, vasodilatation, and increasing vascular permeability. At physiological levels, C5a contributes positively to innate immunity. For example, it acts as a strong chemoattractant for inflammatory cells such as neutrophils, monocytes, and T cells, activates phagocytic cells, and stimulates the release of granule-based enzymes and the generation of reactive oxygen species [241]. Upon binding to its receptor (C5aR/CD88) on neutrophils, C5a induces their transformation into migratory cells capable of infiltrating infected tissue and promoting pathogen clearance [239,242].

However, in case of severe infections, excessive C5a generation during sepsis stimulates inflammation and leads to adverse effects [241,243,244,245,246,247]. C5a was found to contribute to the production of inflammatory mediators (e.g., IL-1β, IL-6, and IL-8) [248,249,250], inhibit phagocytic activity, and impair the neutrophil respiratory burst in a dose-dependent manner [251]. At high doses, C5a also stimulates anti-apoptotic signaling in neutrophils [250,252,253,254], resulting in decreased apoptotic rate and prolonged release of toxic cellular products. Although neutrophil degranulation and liberation of cytotoxic oxygen radicals and other components into extracellular space support pathogen elimination [242,255,256], these molecules can also harm exposed tissue cells. This damage is amplified due to the increase in resistance to apoptosis and sustained presence of neutrophils in tissues. In addition, neutrophils may release their contents as NETs [257,258], a process that may result in neutrophil survival (vital NETs) or death (NETosis). Notably, C5a is about 100 times more potent than C3a.

C5a not only contributes to the development of inflammatory disorders, such as sepsis and acute lung injury caused by bacterial pathogens [246,259], but also contributes to inflammation and acute lung injury in severe viral infections, including influenza, severe acute respiratory syndrome, and COVID-19 [259,260,261,262,263,264].

#### 3.3.3. Nonclinical Studies on the Effects of Attenuation of Anaphylatoxin Release in Disease

In vivo studies have demonstrated that direct blockade of C5a—either by preventing its engagement to C5aR or C5L2, or by blocking its generation—seems highly protective and improves survival in septic animal models. For instance, inhibition of C5a with a monoclonal anti-C5a antibody in a murine CLP model prevented the development of multiorgan failure [147,265] and death [251,266,267,268,269,270]. Similarly, treatment of septic baboons with a C3 convertase inhibitor, which is upstream of C5, protected against *E. coli*-induced organ failure in primates [271,272].

As shown in several studies, polyvalent Ig preparations can also attenuate the generation of anaphylatoxins [10,273,274]. Treatment of CLP-infected septic rats with Ig reduced the anaphylatoxic activity of C5a and prevented neuronal dysfunction in the brain [275]. In this model, an IgM/IgA-enriched Ig showed a more favorable effect on the blood–brain barrier integrity than an IVIg [272]. In a murine asthma model, interaction of the F(ab)_2_-fragment of IgG with anaphylatoxins prevented direct chemotaxis of neutrophils and reduced cellular infiltration into the lung [274]. IgG also blocked the interaction of C5a with endothelial C5aR, preventing the anaphylatoxin-induced anaphylactic shock.

#### 3.3.4. Clinical Studies on Modulation of Complement Activity

Few clinical studies evaluated the effect of IVIg preparations on levels of C3, C4, and anaphylatoxins, albeit for indications other than severe infections [276,277,278]. Only one study investigated the effect of an IgM/IgA-enriched Ig in healthy subjects and patients with sCAP, showing a transient, dose-dependent decrease in C3 and C4 levels [15]. This may indicate enhanced opsonophagocytosis and pathogen clearance.

Despite limited clinical evidence, the importance of complement regulation is supported by findings in a study on patients who died from sepsis [279]. C3a and C4a levels were considerably elevated in these patients, which correlated with disease severity. Similarly, in other prospective studies, high levels of anaphylatoxins were associated with mortality, and it was discussed that complement may play a role in multiorgan failure [279,280]. Surviving septic patients had lower anaphylatoxin levels than non-survivors [281,282]. In a prospective study on patients with sepsis, high C5a levels reduced C5aR and C5L2 expression levels and IL-8 production in neutrophils, which was associated with poor outcomes [147]. In a recent study combining systematic review and meta-analysis with validation using an ICU database and three independent proteomic datasets, it was revealed that sepsis non-survivors exhibited lower C3 and C4 levels and higher C4a, consistent with complement activation and/or depletion [283].

Emerging data suggest that targeting the terminal complement pathway may have clinical benefits. At the time of this review, several monoclonal antibodies and small molecules targeting different components of the complement cascade are being evaluated [284]. One of the most recent developments is a molecule intended to be used in severe infections caused by severe acute respiratory syndrome coronavirus 2.

Whether C5a inhibition alone is sufficient to provide clinical benefit in severe bacterial infections, such as sepsis, or other types of infections remains to be investigated. Similar to other immune-suppressing strategies, the risk of infectious complications may increase. So far, this has not been observed for the immune-modulating and antipathogenic activities provided by polyvalent Igs.

Thus, for this mode of (inter)action, data on complement modulation by Ig in severe bacterial infectious disease is still limited. While in vitro Ig preparations appear to modulate complement activation, the clinical relevance of this mechanism remains speculative. Larger studies are needed to support the role of Ig-mediated complement modulation and to determine whether complement regulation translates into improved outcomes.

### 3.4. Summary of Section 3

**Figure 2:**
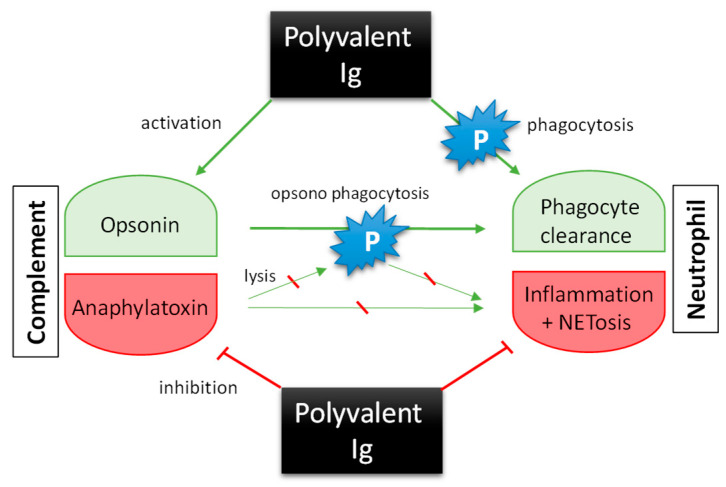
Summary of the interplay between Igs, pathogens, neutrophils, and complement. Green arrows represent interactions that induce the indicated positive effects in severe infections. Red lines represent interactions that inhibit the indicated negative effects that would cause further inflammation in severe infections. See text for further explanations. Ig: immunoglobulin; NET: neutrophil extracellular traps; P: pathogen.

Nonclinical and clinical results suggest that Ig treatment is at least partially effective by immune modulation in patients with severe infections. IgA, IgG, and IgM exert different mechanisms of (inter)action and thus together provide a broad spectrum of modulation. Balancing the immune response is important to prevent excessive overstimulation, hyperinflammation, and immune exhaustion in patients with severe bacterial infections. Ig preparations act on the host to limit these processes by acting on immune cells like neutrophils and on the complement system (Figure 2) in a dual (activating and inhibiting) manner. For example, whereas upregulation of CD64 surface expression on neutrophils— critical for pathogen clearance— increases phagocytic activity, Igs help maintain a balance between their activation and inhibition to avoid exhaustion of immune cells and hypo-reactivity. Particularly, IgM/IgA-enriched preparations limit endotoxin-induced CD64 expression on neutrophils. Similarly, Igs activate complement and enhance pathogen clearance via (opsono)phagocytosis, while also inhibiting CDC and attenuating the release of anaphylatoxins. Finally, complement factors further modulate neutrophil activity (Figure 2). Together, these modes of (inter)action likely play an essential role in limiting inflammation and tissue damage induced by infection, inflammation, or coagulation processes (summarized in Ref. [285]). However, clinical studies are required to further support these hypotheses and expand upon the currently available nonclinical and clinical data.

## 4. Important Lessons Learned from Nonclinical and Clinical Studies

Several studies conclude that the type and dose of Igs are relevant for the effective treatment of sepsis and septic shock [7,8,73,286,287,288]. Two additional, interrelated aspects are highlighted as key concepts.

### 4.1. Timepoint of Immunoglobulin Administration

Timely administration of Igs plays a crucial role in enhancing the efficacy of treatment in severe infections [49,289,290] and has been discussed in several reviews [7,8]. A retrospective study showed that survivors of septic shock caused by MDR bacteria received IgM/IgA-enriched Ig with a shorter delay than non-survivors (median delay: 18 h, n = 25 vs. 66 h, n = 21) [290]. A longer delay was significantly associated with increased risk of in-ICU mortality (odds ratio = 1.007, 95% CI: 1.0006–1.014, *p* = 0.048).

Marked benefits on mortality rates, albeit not always significant due to small sample sizes, were observed when Igs were administered prophylactically (e.g., directly after surgery, diagnosis, or within 24 h of severe sepsis or septic shock) [54,199,206,291,292,293]. Despite a few exceptions [210], no benefit was observed when septic shock was already accompanied by (multiple) organ failure [205,294].

It is thought that the timely clearance of antibody-opsonized pathogens, toxins, and pathogen debris by neutrophils and other phagocytes elicits the beneficial effect of Ig treatment. Impaired neutrophil functions that develop during sepsis can be prevented by Ig, by modulating dysregulated inflammatory response, or by balancing complement activity, particularly C5a, in a timely manner. This implies that administration of Ig is required early after diagnosis to prevent entering such a progressed disease stage. If damage to tissue and multiple organs or coagulopathy have progressed too far, Ig treatment is not able to repair such damage or prevent death (e.g., from thromboembolic events).

### 4.2. Type of Patients

As mentioned before, there is a subgroup of patients with severe sepsis or septic shock that may benefit from the delivery of (high-dose) Ig preparations. However, neutropenic patients may not belong to this target group due to Ig-relevant modes of action related to the interactions with neutrophils [285]. To date, two clinical studies in neutropenic sepsis patients showed no benefit of Ig treatment on mortality rates [55,295].

Therefore, patients eligible for Ig treatment should have an immune system capable of responding to Ig therapy. Additionally, prospective and retrospective studies suggest that patients with moderate disease severity—such as a low SOFA (<8) or a mid-range *Acute Physiology and Chronic Health Evaluation* (APACHE-II) score (~10–25)—may represent a population more likely to benefit from Ig treatment [64,194,201,205,206,210,296,297,298,299]. Patients with very high APACHE-II scores and/or multiple organ failure do not seem to benefit from Ig treatment and may die of other causes [207,208]. Patient selection may also be based on specific biomarkers indicating disease severity [21,181,192,195,196,197,198,203,205,206,208,209,220], on a specific type of pathogen [54,60,62,66,69,122,300,301,302], or immune status at baseline [21,61,69,181,220,295,303,304,305,306,307,308,309,310,311,312]. Table 3 summarizes candidate enrichment strategies, key open questions to guide such future prospective trials, and reference examples that support such strategies or provide further recommendations for such trials with Ig.

### 4.3. Limitations and Safety Considerations

Despite the promising mechanistic rationale, clinical evidence supporting the different mechanisms of action of Ig therapy in adult severe bacterial infectious diseases remains limited. Key limitations of the studies evaluated include the fact that most trials have used hard endpoints like mortality, instead of mode-of-action-related endpoints, which are further downstream, where detection becomes more difficult in a heterogeneous disease population with sepsis. Furthermore, timing, as well as the type of Ig, is important, as not all Ig products are mechanistically equivalent and work with the same efficacy if given at any timepoint of disease. Even though Ig therapy likely works through multiple real mechanisms, efficacy depends on the right patients, at the right time, with the right product—and current trials (and meta-analyses) have rarely aligned all factors.

In addition to the fact that clinical readouts have been poorly matched to biology, most trials were done with small sample sizes, not powered to show efficacy, and additionally conducted within a very heterogenic patient population, or they were retrospectively designed. These factors likely explain why single trials did not always show clear benefits. Therefore, pooled analyses have been more successful in showing which patient cohorts most likely benefit from a specific Ig type, and with what dose. But also here, most have relied on hard endpoints so far, and have rarely included, e.g., mechanistic biomarkers, pathogen-specific outcomes, toxin activity, or immune function readouts. Pooled analyses test for clinical association rather than mechanistic validity.

Another important limitation of this review is that the nonclinical and clinical evidence discussed spans a period of more than four decades. During this time, not only have standards of care evolved, but so have the production processes of Ig preparations, the available antibiotic repertoire, and overall sepsis management. Furthermore, early studies have applied heterogeneous protocols, including the use of different types of Ig preparations, as well as variability in the timing of administration and dosing regimens. These differences limit the comparability of individual studies and complicate the extrapolation of historical findings to contemporary clinical practice and the design of future trials.

Limitations on the use of Ig are also related to the fact that Ig administration is accompanied by clinical challenges. Although Ig treatment is generally well-tolerated, several adverse effects have been reported more frequently, particularly the risk of infusion reactions, e.g., related to infusion rate, osmolarity of the product, and sodium content. Close monitoring of patients during infusion is highly relevant. However, the interpretation of adverse events in this setting is inherently challenging. On the one hand, IVIg-associated risks—including thromboembolic events, renal dysfunction, and hemolysis—occur in treated patients with sepsis or septic shock. On the other hand, this patient cohort frequently presents organ dysfunction and multiple pre-existing risk factors. In addition, complications such as acute kidney injury or thromboembolism are also common consequences of the underlying disease. Moreover, safety profiles may vary depending on immunoglobulin formulation (e.g., excipients, stabilizers, IgA, or anti-A/B content), manufacturing processes, dosing, timepoint of administration, infusion rate, and patient-specific factors [326,327,328,329,330,331]. As reporting of safety data from clinical studies is still limited, standardized reporting of adverse events from randomized placebo-controlled trials is important to better define the benefit–risk profile of different immunoglobulin preparations in specific septic populations.

## 5. Conclusions and Future Directions

Ig administration provides a broad spectrum of antibody-binding activity to PAMPs and DAMPs, helping to improve clearance by phagocytic cells. Considering the growing antibiotic resistance of bacteria, this type of therapy may provide protection against infection through mechanisms that are not impacted by resistance. Furthermore, the presence of IgM and IgA in polyvalent Ig preparations further enhances interactions with complement factors and reduces the release of cytokines by neutrophils, thereby more effectively dampening inflammatory processes than IVIg. The pharmacologic rationale for treating severe bacterial infections such as sepsis with Ig preparations is supported by observations of low endogenous Ig levels (mostly IgM and IgG) in affected patients and the clinical benefits seen in these cases [303,306,312,315,332,333].

Although all this is clear, the clinical evidence generated in the past for Ig use in severe bacterial infections has been rated “weak” so far. However, more recent studies and meta-analysis in specific patient populations seem supportive for IgM/IgA-enriched Ig and high-dose Ig preparations [287,297,334,335,336,337,338].

Based on the understanding of the molecular mechanisms of Ig preparations from previous nonclinical and clinical studies, this review aimed to highlight the importance of defining more suitable target populations most likely to benefit from Ig treatment. Eligible patients should present clear signs of infection (avoiding vague terms like “suspected infection”) or have a specific type of pathogen (e.g., Gram-negative sepsis, STSS, nosocomial infection). Selection may in the future also be based on low endogenous Ig levels at ICU admission or on target Ig levels to be reached after treatment [307,321]. Previous studies also underscore the importance of appropriate antibiotic use to observe any additive effects and to eliminate the chance of patients dying from inappropriate antibiotics rather than from inappropriate Ig treatment. Timing of Ig administration and disease severity are closely related and should be carefully considered when planning future studies. Finally, to draw meaningful conclusions, the number of patients planned to be enrolled should, whenever possible, be based on sample size calculations.

Future research should prioritize early intervention, biomarker-guided patient selection, adequately powered randomized trials, and the integration of secondary efficacy and pharmacodynamic endpoints that are related to the postulated mechanisms of action. This would add to a better understanding of how Ig products benefit which patients most.

In view of the marked heterogeneity of sepsis, and the complex modes of (inter)actions of Ig, advanced analytical approaches, including machine learning- and artificial intelligence-based methods, may further support future trial design. The integration of previously generated evidence and detailed knowledge on the modes of actions of Ig, together with clinical characteristics, biomarker profiles, and immunological parameters of the patients treated, may well help to establish personalized Ig therapy in sepsis, achieving optimal efficacy in the future.

## Figures and Tables

**Table 1:** Characteristics of polyvalent immunoglobulins and polyvalent immunoglobulin preparations.

	Polyvalent IgM (Immune [Adaptive] IgM; Natural IgM) ^3^	Polyvalent IgA (Isotypes: IgA1, IgA2) ^4^	Polyvalent IgG (Isotypes: IgG1, IgG2, IgG3, IgG4) ^5^
**Characteristics of Ig preparations**			
IVIg products	<0%	<1%	≥95%
Pentaglobin ^1^	12%	12%	76%
Trimodulin ^2^	~23%	~21%	~56%
**Characteristics of Igs**			
Structure	Pentameric IgM: associated with J-chain, 10 antigen binding sites Hexameric IgM: no J-chain, 12 antigen binding sites	Monomeric IgA1 and allotypes IgA2m(1), IgA2m(2), IgA2m(n): 2 antigen binding sites Dimeric IgA: associated with J-chain, 4 antigen binding sites SIgA: associated with J-chain and secretory component Multimeric IgA: >6 antigen binding sites	Monomer: 2 antigen binding sites
Plasma level	~6% (0.4–2.3 g/L)	~13% (0.7–4 g/L)	~80% (7–16 g/L)
Half-life	5 days	6 days	20–25 days
Location	Plasma, mucosa, interstitial fluid	Monomeric IgA1: main IgA isotype in plasma SIgA dimers or multimers: transport across epithelium to mucosa, saliva, tears (IgA1, IgA2)	Plasma, interstitial fluid
Main biological functions	Immune IgM: primary response against invaders, produced by B2 cells Natural IgM: tissue homeostasis, removal of apoptotic/dead cells, constitutively produced by B1 cells IgM: complement fixation Monomeric IgM: B cell receptor Anti-inflammatory activity (modulation of cytokine responses) Regulation of immunity and tolerance Modulation of immune cell functions	Secretory antibody for pathogen protection (via neutralization and immune exclusion) at mucosa (e.g., lung) and epithelium (e.g., gut) IgA1: pathogen clearance in blood IgA immune complexes trigger FcαRI to synergize with various other receptors to amplify pro-inflammatory responses Soluble monomeric and dimeric IgA, predominantly anti-inflammatory activity (modulation of cytokine responses) Modulation of immune cell functions	Main blood antibody Long-term immunity and memory Opsonization, clearance of pathogens Neutralization of toxins Only immunoglobulin class in neonates (maternal IgG can cross the placenta) Anti-inflammatory activity (modulation of cytokine responses, binding to anaphylatoxins) Modulation of immune cell functions
Fc domain: binding and function	Binds to Fcµ receptor on B-, T-, and NK-cells and Fcα/µ (CD351) or APC Binds to polymeric Ig receptor for transport across epithelial barrier	Binds to Fcα receptor (CD89) on myeloid immune cells (induction of degranulation, phagocytosis, chemotaxis, ADCC) Binds to polymeric Ig receptor for transport across epithelial barrier	Binds to Fcγ receptors (CD64) on neutrophils, monocytes, granulocytes, eosinophils, macrophages, B-, and NK-cells (induction of phagocytosis, ADCC)
Fab domain: Antigen binding strength	High avidity	High specificity and affinity	High specificity and affinity
Pathogen clearance (neutralization)	Pathogen clearance by binding endotoxin and complement (opsonophagocytosis) Agglutination of antigens Induction of CDC	Phagocytosis Induction of ADCC	Pathogen clearance via phagocytosis Neutralization of exotoxin Induction of CDC and ADCC
Response time	Early response (1–2 weeks)	Early response (1–2 weeks)	Late response (3–4 weeks)
Complement interactions	Strongest activator of the classical pathway IgM > IgG3 > IgG2, IgG1 Scavenging of opsins (C3b, C4b) Concentration-dependent inhibition of CDC	Activator of the alternative pathway Activator of the lectin pathway	IgG3 < IgG1 and 2: activator of the classical pathway IgG3: activator of the alternative pathway Binding to anaphylatoxins (C5a, C3a) Scavenging of opsins C3b, C4b (but: IgM > IgG)

^1^ Also referred to as IgM-enriched preparation, IgGAM. ^2^ Also referred to as IgM Concentrate, BT086, BT588. ^3^ Data obtained from Basta [10], Keyt et al. [14], and Schmidt et al. [15]. ^4^ Data obtained from Bakema et al. [9], Breedveld et al. [12], and van Gool et al. [16]. ^5^ Data obtained from Bournazos et al. [11], Durandy et al. [13], and Vidarsson et al. [17]. ADCC: antibody-dependent cell-mediated cytotoxicity; APC: antigen-presenting cell; C3a: complement factor 3a; C3b: complement factor 3b; C4b: complement factor 4b; C5a: complement factor 5a; CD: cluster of differentiation; CDC: complement-dependent cytotoxicity; Fc: fragment crystalline; Fab: antigen binding fragment; Ig: immunoglobulin; IVIg: intravenous immunoglobulin; NK: natural killer; SIgA: secretory immunoglobulin A.

**Table 2:** Summary of in vitro studies on the neutralizing activity of immunoglobulin preparations against bacterial exotoxins.

Exotoxin Class Name	Source	Ig Preparation	Activity Description	References
**Superantigens ^1^**				
SEC1, SEC2, SEC3, SEE, SE-A, Exfol, SE-B, TSST-1, Supernatants	*S. aureus*	IVIg	High antibody titers against the different toxins were present in IVIg. IVIg inhibited in vitro stimulation of human T cells by the various Staph-toxins. However, in human serum, IgM antibodies target TSST-1. Of the Ig preparations, only IgM/IgA-enriched Ig had powerful activity against TSST-1.	[90,98,101]
SpeA, SpeB, SpeF, SmeZ, SEA, Supernatants	*S. pyogenes*	IVIg, IgM/IgA-enriched Ig, IgAbulin, IgM (Hu299), purified IgA	Ig preparations inhibited Spe-stimulation of mononuclear cells and reduced lymphokine and monokine production. In *S. pyogenes*-infected mice, IVIG neutralized circulating superantigens and reduced systemic inflammation. Ig types neutralized different toxins with different levels of activity.	[49,91,92,96,97,98,101]
GAS supernatant	*Group A streptococcal (GAS) isolates*	IVIg	Infusion of IVIg in patients with GAS infection (STSS and necrotizing fasciitis) neutralized the superantigenic activity, produced by their respective GAS isolate	[92,96,98]
**Membrane-damaging or pore-forming toxins ^2^**				
α-toxin, α-hemolysin, PVL, Coagulase, Supernatants	*S. aureus (incl. MRSA)*	IVIg, IgM/IgA-enriched Ig	IVIg significantly reduced the hemolytic and coagulase activity of MRSA. Two specific IVIg antibodies neutralized the toxic effects of α-hemolysin and PVL and conferred protection against necrotizing pneumonia in the rabbit model. IgG reactivity against α-toxin was detectable in both preparations. Also, IgM reactivity was detected against α-hemolysin. IgM/IgA-enriched Ig provided higher protection against G^pos^ infection in mice compared to IVIg. The *S. aureus* supernatant was more effectively neutralized by the IgM containing Ig preparation.	[33,35,93,94,97]
Streptolysin	*S. pyogenes*	IVIg, IgM/IgA-enriched Ig	IgG reactivity against streptolysin was detectable in both preparations.	[35]
Pneumolysin	*S. pneumoniae*	IVIg, IgM/IgA-enriched Ig	Both Ig preparations efficiently inhibited pneumolysin-induced platelet destruction. The capacity to antagonize pneumolysin mainly depended on the final IgG content.	[99,100]
**A-B toxins ^3^**				
PcrV, ETA, Supernatants	*P. aeruginosa*	IVIg, IgM/IgA-enriched Ig	Anti ETA-IgG from human serum had a prophylactic and protective effect in *P. aeruginosa* pneumonia and ETA toxemia models. Anti-PcrV inhibits toxin translocation into the cells and is present in IVIg. IVIg prevented AKI and mortality in *P. aeruginosa*-infected mice. IgM/IgA-enriched Ig provided better neutralization in vitro and higher protection against G^neg^ infection and toxin-related lethality in mice compared to IVIg.	[33,87,88,89,95]

^1^ Cause extensive T-cell proliferation (mitogenic activity) by cross-linking MHC class II molecules on APCs with the TCR, leading to a massive cytokine storm leading to fever (pyrogenic activity), rash, and toxic shock. ^2^ Cause pore formation or enzymatic membrane destruction, leading to cell lysis and tissue necrosis. ^3^ Inhibit intracellular protein synthesis and signaling by binding of the B subunit to the host cells, and the A subunit to EF-2. AKI: acute kidney injury; ETA: exotoxin A; Exfol: exfoliative toxin; G^neg^: Gram-negative; G^pos^: Gram-positive; PcrV: mnemonic used for different bacterial effector functions (pore-forming, cytoskeletal disruption, ribosylation, and vesicle-trafficking inhibition); PVL: Panton–Valentine leucocidin; SE-A: staphylococcal enterotoxin A; SE-B: staphylococcal enterotoxin B; SEC: staphylococcal enterotoxin C; SEE: staphylococcal enterotoxin E; SmeZ: streptococcal mitogenic exotoxin Z; SpeA: streptococcal pyrogenic exotoxin A; SpeB: streptococcal pyrogenic exotoxin B; SpeF: streptococcal pyrogenic exotoxin F; STSS: streptococcal toxic shock syndrome; TSST: toxic shock syndrome toxin-1.

**Table 3:** Proposed patient enrichment strategies for immunoglobulin therapy in severe bacterial infections.

Proposed Enrichment Markers for Future Trials	Open Questions	Supporting References
Time window of success: - Early administration (e.g., within 24 h of sepsis onset, diagnosis of septic shock, or requirement of IMV)	- What is the optimal start point and time window for Ig administration relative to symptom onset? At sepsis diagnosis, at ICU admission, or at start of organ support? - Is optimal timing dependent on Ig type or dose?	Prospective: [21,54,200,201,294,313] Retrospective: [290,292,314] Review on further recommendations: [288]
Organ dysfunction status: - Low or mid-range SOFA - Mid-range APACHE - Low coagulopathy score (e.g., DIC)	- What score range best identifies patients with potentially reversible organ dysfunctions who would benefit from supplemental Ig treatment? - Is a faster decrease in the score/faster recovery related to the Ig levels reached? - Do low Ig baseline levels correlate with score levels? - Are SOFA/APACHE score ranges consistent across infection types and Ig products?	Prospective: [64,194,201,205,206,207,296,297] Retrospective/post hoc: [194,210,297,298,299] Review on further recommendations: [202]
Immune status: - Low IgG baseline and/or low IgM baseline - Lymphopenia - Low HLA-DR on MNs - Low CD64 on PMNs/MN - Neutropenia	- Which immune dysfunction markers best define the patients that respond best to Ig treatment? - Can Ig (faster) restore immune dysfunction and what Ig thresholds are to be reached? - Which level of immune dysfunction markers is best related to the time window of success for Ig treatment? - Is Ig treatment most effective if the type of Ig is based on the specific Ig deficiency at baseline? - Which markers reflect treatable immune dysfunction rather than a stage of late immunosuppression? - Can emerging immune phenotyping approaches be incorporated in clinical trial design to assess functional status?	Prospective: [61,220,295,303,304,305,306] Observational: [69,307,308,309] Retrospective/post hoc: [21,181,310,311,312] Meta-analysis [315] Review on further recommendations: [8,202,316,317,318]
Inflammatory profile: - High CRP, PCT, and/or IL-6 levels - High neutrophil counts or neutrophil-related markers like NETosis markers or NLR	- Can Ig reduce level of hyperinflammation and is there a correlation between % decrease and % survival? - Which inflammatory biomarker patterns reflect treatable hyperinflammation? - Can inflammatory markers improve treatment timing and patient selection?	Prospective: [192,195,196,203,205,206,208,220] Retrospective/post hoc: [21,181,197,198,209] Review on further recommendations: [202]
Etiology: - Gram-negative sepsis - STSS - MDR/XDR - Hospital vs. community-acquired infections	- Is Ig therapy particularly effective in a specific type of infection? - Is endotoxin neutralization dependent on the type of Ig preparation? - Is there a correlation between level of endotoxin neutralization, time to recovery, and mortality? - Can registry- or KI-supported retrospective trials be helpful in investigating Ig efficacy in specific types of infection and preventing issues with large sample sizes and slow recruitment in prospective trials?	Prospective: [54,62,66] Comparative/observational: [69,300] Retrospective/post hoc: [60,122,301,302] Meta-analysis/review: [112] Review on further recommendations: [319,320,321,322,323,324,325]

Supporting references may be from studies showing efficacy, as well as from negative/neutral studies demonstrating that the selected patient population in such studies did not benefit from Ig treatment with the administered type of Ig and dose. APACHE: Acute Physiology and Chronic Health Evaluation; CD: cluster of differentiation; CRP: C-reactive protein; DIC: disseminated intravascular coagulation; HLA-DR: human leukocyte antigen—DR isotype; ICU: intensive care unit; Ig: immunoglobulin; IL: interleukin; IMV: invasive mechanical ventilation; KI: knowledge integration; MDR/XDR: multidrug-resistant/extensively drug-resistant; MNs: mononuclear cells; NETosis: neutrophil extracellular trap formation; NLR: neutrophil-to-lymphocyte ratio; PCT: procalcitonin; PMNs: polymorphonuclear neutrophils; SOFA: Sequential Organ Failure Assessment; STSS: streptococcal toxic shock syndrome.

## Data Availability

No new data were created or analyzed in this study.

## Supplementary Materials

The following supporting information can be downloaded at https://www.mdpi.com/article/10.3390/biomedicines14020399/s1, Table S1: Clinical studies investigating effects of immunoglobulins on the pathogens and their toxins; Table S2: Clinical studies investigating effects of immunoglobulins on the host.
