# Supplementary material for: Modes of (Inter)Actions of Polyvalent Immunoglobulins: Nonclinical and Clinical Research in Severe Bacterial Infections
Source: Biomedicines. 2026 Feb 9;14(2):399. doi: 10.3390/biomedicines14020399 (PMC12938816; doi:10.3390/biomedicines14020399)
Supplement: Supplementary file 1 [file biomedicines-14-00399-s001.zip › biomedicines-4082743-supplementary/Supplementary Table S1_06Feb2026.pdf]

Modes Of (Inter)actions of Polyvalent Immunoglobulins: Nonclinical and Clinical Research in Severe Bacterial Infections

Sabrina Weißmüller [1,2], Carolin Schmidt [3,4] and Corina Heinz [3,*]

Supplementary Materials

Table S1. Clinical studies investigating effects of immunoglobulins on the pathogens and their toxins

References	Indication	Ig preparation<br>(Dose)<br>Total dose[1]	N	Effect	Significant	Marked change	No difference	Study Results
Schedel et al. (1991) [54]	Septic shock	IgM/IgA-enriched Ig (600 mL on day 1 and 300 mL on day 2 to 3)<br>**Total Ig dose:**<br>(1200 mL)<br>~800 mg/kg BW[1]	55	- EA neutralization<br>- Mortality	X<br>X			• Facts:<br>o Endotoxin concentration: Significantly increased in the first 48 h after sepsis onset.<br>o IgG titers against lipid A: Decreased in non-survivors.<br>o Endotoxin concentration at 24 h: Significantly lower in survivors vs non-survivors ($p < 0.01$).<br>• Endotoxin levels at 24 h: Significantly reduced in Ig group ($p < 0.01$).<br>• Mortality: 4% (1/27) in Ig group vs 32% (9/28) in control ($p < 0.01$).
Behre et al. (1992) [55]	G^{neg} sepsis or septic shock in patients with severe neutropenia and hematologic malignancy	IgM/IgA-enriched Ig (200 mL loading dose followed by 100 mL every 6 hours for 72 hours)<br>**Total Ig dose:**<br>(1400 mL)<br>~933 mg/kg BW[1]	52	- EA neutralization<br>- Mortality	X	 X		• Facts:<br>o Mortality in endotoxin-positive sepsis: 41% (7/17); with septic shock: 64% (7/11).<br>o All endotoxin-negative patients (n=4) survived.<br>o Endotoxin concentration correlated with mortality ($p < 0.05$).<br>• Ig infusion. Significantly increased antibodies against endotoxin.<br>• Overall mortality: 30% (9/30) in Ig group vs 45% (10/22) in control ($p > 0.05$, n = 52).<br>• Mortality in participants with septic shock at baseline: 54% vs 69% ($p > 0.05$).

References	Indication	Ig preparation (Dose) Total dose[1]	N	Effect	Significant	Marked change	No difference	Study Results
Wand et al. (2016) [56]	Severe sepsis	IgM/IgA-enriched Ig (250 mg/kg over 12 h on 3 days) **Total Ig dose:** 750 mg/kg BW	26	- EA neutralization	X			• Endotoxin concentration: Significantly decreased in Ig group after 6 and 12 h of treatment (p <0.05).<br>• Endotoxin concentration: Significant lower in Ig group vs control group after 6 h (p <0.05).
Ziegler et al. (1991) [57]	G^{neg} bacteremia and septic shock	Anti-endotoxin monoclonal IgM antibody (1 dose of 100 mg HA-1A antibody) **Total MAB dose:** ~1.3 mg/kg BW[1]	200 G^{neg} 543 in total	- Mortality (G^{neg})<br>- Mortality (All)	X 	 X		• Mortality in sepsis/bacteremia: Significantly reduced in HA-1A group (32/105) vs control (45/92) (p = 0.014).<br>• Mortality in participants with septic shock at baseline: 18/54 vs 27/47 (p = 0.017).<br>• No benefit of HA-1A in 343 patients without proven G^{neg} sepsis.<br>• Overall mortality: 39% vs 43% (p = 0.24, n=543).
Bone et al. (1995) [58]	G^{neg} sepsis	Anti-endotoxin monoclonal IgM antibody (2 doses of 2 mg E5/kg/day) **Total MAB dose:** 4.0 mg/kg BW	847	- Mortality<br>- MOF resolution<br>- MOF prevention	 X<br>X		X	• Survival: No significant improvement in E5 group vs placebo in G^{neg} sepsis (p = 0.21, n = 530).<br>• Survival: No benefit of E5 in G^{neg} sepsis + organ failure (p = 0.3, n = 139).<br>• MOF resolution: Significantly higher in E5 group vs control (48% vs 27%, n = 139), regardless of etiology.<br>• E5 significantly prevented ARDS development (p = 0.007).<br>• E5 reduced nervous system dysfunction (p = 0.050).
Kaul et al. (1999) [69]	STSS	IVIg (median cumulative high dose: 0.4 g/kg for 5 days) **Total Ig dose:** 2000 mg/kg BW	44	- T-cell mitogenicity<br>- Survival	X<br>X			Fact: Superantigens (enterotoxins, TSS toxin-1, SpeA) induce massive T-cell proliferation and cytokine production.<br>• Mitogen-neutralizing activity: Significantly increased in 10 patients of Ig group after first or second dose.<br>• Survival: Ig group (n = 21), 90% on day 7 and 67% on day 30 vs controls 50% (p <0.01) and 34% (p = 0.02), respectively.
Darenberg et al. (2003) [62]	STSS	IVIg (1 g/kg day 1 and 0.5 g/kg day 2 to 3) **Total Ig dose:** 2000 mg/kg BW	21	- Mortality<br>- MOF	 X	X 		• Superantigen-neutralizing activity: Significantly higher in Ig group on treatment days 2 (p = 0.003) and 3 (p = 0.04).<br>• Mortality: 3.6-fold lower in Ig group vs placebo (NS, p = 0.3).<br>• SOFA score: Reduction significantly higher in Ig group vs placebo on days 2 and 3 (p = 0.02 and p = 0.04).

References	Indication	Ig preparation (Dose)<br>Total dose[1]	N	Effect	Significant	Marked change	No difference	Study Results
Borleffs et al. (1993) [59]	Persistent *C. jejuni* infection in immune-compromise patients	IVIg<br>(6 doses 300 mg/kg every 3 weeks)<br>**Total Ig dose:**<br>1800 mg/kg BW<br>IgM/IgA-enriched Ig<br>(6 doses 350 mg/kg every 3 weeks)<br>**Total Ig dose:**<br>2100 mg/kg BW	2	- SBA		X		• Facts:<br>o In hypogammaglobulinemic patients, C. jejuni infections can relapse and cause systemic infection (bacteremia).<br>o Two patients received antibiotics and Ig but still had recurrent infections despite adequate IgG levels.<br>• SBA: Increased to 90% 1h after IgM/IgA-enriched Ig treatment; no culture-proven relapse occurred.<br>• SBA remained elevated until next IgM/IgA-enriched Ig dose.
Busani et al. (2019) [60]	Septic shock caused by MDR pathogen	IgM/IgA-enriched Ig<br>(250 mg/kg on 3 days)<br>**Total Ig dose:**<br>750 mg/kg BW	94	- Mortality from MDR infection	X			• Fact: History of cancer and MDR *A. baumannii* infection increase mortality risk in patients with septic shock.<br>• Mortality risk: Significantly reduced after Ig treatment (OR = 0.28; 95% CI: 0.14-0.59; $p = 0.001$).<br>• 30-day mortality: 31.2% in Ig group (n = 46) vs 54.3% in control (n = 48) ($p = 0.02$).
Giamarellos-Bourboulis et al. (2016) [66]	Severe sepsis or septic shock caused by G^{neg} MDR and XDR pathogen	IgM/IgA-enriched Ig<br>(mean dose: 30 g/day for 5 days)<br>**Total Ig dose:**<br>1500 mg/kg BW	200<br>(113 severe sepsis, 78 septic shock)	-Overall mortality from MDR/XDR infection<br>-Mortality from XDR infection<br>-Duration of sterile blood culture >72h<br>-BT Bacteremia<br>-Time to BT bacteremia	X<br>X	X<br>X<br>X		• 28-day mortality: 39/100 in Ig group vs 58/100 matched comparators ($p = 0.011$).<br>• Most survival benefits in patients with cardiovascular failure (41.9% in Ig group vs 62.8% in comparators, $p = 0.004$) and patients with XDR G^{neg} infections (38.5% in Ig group vs 62.9% in comparators, $p = 0.008$).<br>• Sterile blood cultures >72h: 65 patients vs 30 patients.<br>• Median BT bacteremia time: within 10 days in 22/65 (33.8%) in Ig group vs within 4 days in 12/30 (40.0%) in comparators ($p = 0.682$).
Grundmann et al. (1988) [67]	Post-operative G^{neg} sepsis and endotoxemia	IVIg<br>0.25 g/kg on day of study entry and the following day.<br>**Total Ig dose:**<br>500 mg/kg BW	74	-EA neutralization<br>-Mortality	X	X		The endotoxin course was measured in patients with postoperative sepsis (n = 46):<br>• Endotoxin-positive patients: Significantly decreased in Ig group (~30%) vs control (~70%) ($p < 0.02$).<br>• Mortality: 63% (15/24) in Ig group vs 83% (19/22) in control (NS).

References	Indication	Ig preparation (Dose) Total dose[1]	N	Effect	Significant	Marked change	No difference	Study Results
Masaoka et al. (2000) [70]	Sepsis or suspected sepsis, severe infections not responding to broad spectrum antibiotics	IVIg<br>(5 g daily for 3 days)<br>**Total Ig dose:**<br>~200 mg/kg BW[1]	504	-Bacteria eradication<br>-Defervescence<br>-Clinical cure<br>-Decrease CRP<br>-Response rate	 <br><br><br>X	X<br>X<br>X<br>X		• Bacterial eradication: 67.9% (19/28) in Ig group vs 50% (8/16) in controls (p = 0.24).<br>• Afebrile within 7 days: 54.8% vs 37.2%.<br>• Clinical symptoms resolved by day 7: 57.3% vs 39.4%.<br>• CRP levels: Decreased more in Ig group (p = 0.11).<br>• Overall response rated "excellent" or "good": 61.5% (163/265) vs 47.3% (113/239) (p <0.001).
Rodriguez et al. (2005) [71]	Severe sepsis and septic shock of intra-abdominal origin admitted to the ICU within 24 h after the onset of symptoms, post-surgery	IgM/IgA-enriched Ig<br>(7 mL/kg for 5 days)<br>**Total Ig dose:**<br>1750 mg/kg BW	56 (36 severe sepsis, 20 septic shock)	-Overall mortality<br>-Additive effects<br>-Additive survival with Ig + AB	 X<br>X	X		• Fact: Mortality in patients with severe sepsis was 25%, 55% in septic shock and 37.5% overall. Adequate AB was associated with 76.7% survival<br>• Overall mortality: 27.5% in Ig group vs 48.1% in control (p = 0.06).<br>• Mortality with adequate AB therapy: 8.7% in Ig group vs 33.3% in control (p = 0.04).<br>• Survival with adequate AB therapy: >90% with adjunctive high doses of Ig (p = 0.02).
De Simone et al. (1988) [63]	Severe sepsis (G^{pos} and G^{neg}) and septic shock	IVIg<br>(0.4 g/kg on day after admission, 0.2 g/kg after 48 h and 0.4 g/kg 5 days later)<br>**Total Ig dose:**<br>1000 mg/kg BW	24	-Additive survival with Ig + AB<br>-Defervescence time<br>-Bacteria eradication<br>-% days of AB use	 <br>X<br>X<br><br>X	X		• Severe sepsis survival: 42% with Ig + AB vs 25% with AB alone (NS).<br>• Time to afebrile: 10 days in the Ig group vs >16 days in non-Ig group (p <0.01).<br>• Bacterial eradication: 40% in Ig group vs 8% in non-Ig group (p <0.01).<br>• Proportion of days on AB during ICU stay: 38% vs 95% (p <0.01).

References	Indication	Ig preparation<br>(Dose)<br>**Total dose[1]**	N	Effect	Significant	Marked change	No difference	Study Results
Dominioni et al. (1991) [65]<br>Dominioni et al. (1996) [64]	Surgical patients with sepsis scores of 17-30	IVIg<br>(0.4 g/kg on days 0 and 1 and 0.2 g/kg on day 5)<br>**Total Ig dose:**<br>1000 mg/kg BW	113	-Overall mortality	X			• Overall mortality: 33% in Ig group vs 64% in control ($p < 0.005$).<br>• Incidence of fatal septic shock: 7% in Ig group vs 29% in control ($p < 0.01$).<br>• Mortality in patients with intermediate sepsis scores (20-25): 33% in Ig group vs 66% in control ($p < 0.025$).<br>• IgG levels significantly increased from baseline in survivors vs non-survivors ($p < 0.05$), suggesting compensation of high IgG consumption in sepsis by Ig treatment.
				-Mortality from septic shock	X			
				-Mortality from MOF			X	
				-ICU stay of survivors		X		
				-IgG increase in survivors	X			
Cafiero et al. (1992) [61]	Patients at risk for postoperative sepsis after surgery for colorectal cancer	IVIg<br>(15 g if ≥40 kg BW;<br>12 g if <40 kg BW, on day before operation and on day 1 and 5 post-operation.<br>**Total Ig dose:**<br>600 mg/kg BW[1]	80	-Additive prevention of infection in Ig + AB	X			• Postoperative infections: Significantly lower in Ig + AB group (no infection 23/43 [53.5%]) vs AB alone (8/37 [21.6%], $p < 0.001$).<br>• Postoperative IgG level: Significantly higher with Ig + AB vs AB alone (with infection: 1009 vs 817 mg/dL; without infection: 1350 vs 979 mg/dL, $p < 0.01$).<br>• IgG levels: Significantly lower in patients who developed infections in both groups ($p \leq 0.01$).<br>• IgG threshold may discriminate septic complications (32% if >1000 mg IgG/dL and 100% if <1000 mg/dL).<br>• None of the 80 patients died.
				-IgG increase	X			
Wesoly et al. (1990) [72]	Postoperative sepsis	IgM/IgA-enriched Ig<br>5 mL/kg BW for 3 days<br>**Total Ig dose:**<br>750 mg/kg BW	35	-EA neutralization		X		• Endotoxin plasma titers: Decreased on day 5 in Ig group.<br>• Mortality: 44.4% in Ig group vs 76.5% in control.<br>• Duration of hospitalization: 13.3 ± 5.8 in Ig group vs 15.8±7.1 days in control.<br>• Duration of IMV: 9.9 ± 6.6 in Ig group vs 12.8 ± 6.3 days in control.
				-Mortality		X		
				-LOS		X		
				-VD		X		
Just et al. (1986) [68]	Severe bacterial infections after surgery, trauma or intoxication	IgM/IgA-enriched Ig<br>(400 mL in 36 h)<br>**Total Ig dose:**<br>~266 mg/kg BW[1]	104	- Mortality	X			• Fact: Of 104 patients, 29 had sepsis and 64 had pneumonia at baseline. Infections caused by G^{pos} and G^{neg} pathogens in similar proportions.<br>• Mortality risk from infection: Significantly reduced in Ig + AB ($p < 0.05$)<br>• Duration of IMV: Significantly reduced in Ig + AB (5.5 days) vs AB alone (12.7 days, $p < 0.01$)<br>• ICU stay: Significantly reduced (14.8 vs 21.5 days, $p < 0.01$).<br>• Infection course significantly improved in patients with sepsis and pneumonia.
				- ICU stays	X			
				- VD	X			

[1] Total dose was calculated assuming a mean body weight value of 75 kg in case total infusion volume was provided, but the mean weight of the patients was missing.

AB; antibiotic(s); ARDS: acute respiratory distress syndrome; BT: breakthrough; BW: body weight; CI: confidence interval; CRP: C-reactive protein; EA: endotoxin activity; G^{neg}: Gram-negative bacteria; G^{pos}: Gram-positive bacteria; h: hours; ICU: intensive care unit; Ig: immunoglobulin; IMV: invasive mechanical ventilation; IVIg: intravenous immunoglobulin; LOS: length of stay; MAB: monoclonal antibody; MDR: multidrug-resistant; MOF: multiple organ failure; n: number of study patients; NS: not statistically significant; OR: odds ratio; SBA: serum bactericidal activity; SOFA: Sequential Organ Failure Assessment; SpeA: Streptococcal pyrogenic exotoxin A; STSS: Streptococcal toxic shock syndrome; TSS: toxic shock syndrome; VD: ventilated days; XDR: extensively drug-resistant.
