# Supplementary material for: Modes of (Inter)Actions of Polyvalent Immunoglobulins: Nonclinical and Clinical Research in Severe Bacterial Infections
Source: Biomedicines. 2026 Feb 9;14(2):399. doi: 10.3390/biomedicines14020399 (PMC12938816; doi:10.3390/biomedicines14020399)
Supplement: Supplementary file 1 [file biomedicines-14-00399-s001.zip › biomedicines-4082743-supplementary/Supplementary Table S2_06Feb2026.pdf]

Modes Of (Inter)actions of Polyvalent Immunoglobulins: Nonclinical and Clinical Research in Severe Bacterial Infections

Sabrina Weißmüller [1,2], Carolin Schmidt [3,4] and Corina Heinz [3,*]

Supplementary Materials

Table S2. Clinical studies investigating effects of immunoglobulins on the host

References	Indication	Ig preparation (Dose) Total Ig dose[1]	N	Effect	Significant	Marked change	No difference	Study Results
Kaul et al. (1999) [69]	STSS (clinical diagnosis pneumonia or fasciitis)	IVIg (median cumulative dose: 400 mg/kg on 5 days) **Total median dose**: 2000 mg/kg BW	44	- T-cell activation<br>- INFM<br>- Survival	X<br>X<br>X			• Mitogen-neutralizing activity: Significantly increased in 9/10 patients treated with Ig after first or second dose.<br>• IL-6 and TNF-α levels: Significantly decreased in 4/4 patients; and 3/4 patients, respectively (p <0.5).<br>• 7- and 30-days survival of 21 Ig-treated patients: 90% and 67% in Ig group vs 50% and 34% in control, respectively (p <0.01 and p = 0.02).
Ishikura et al. (2015) [197]	Sepsis	IVIg (~80-100 mg/kg for 3 days) **Total Ig dose:** ~240-300 mg/kg BW	41	- INFM<br>- Mortality	X	 X		• Fact: On admission, PCT and IL-6 were significantly higher and IgG significantly lower in Ig group (n = 19) vs control (n = 22).<br>• CRP, PCT, and IL-6 levels during treatment: Significantly decreased from admission in Ig group (all p <0.01). In control group, only IL-6 decreased significantly (p <0.01).<br>• 28-day mortality: 5.3% in Ig group vs 18.2% in control (NS).
Ikeda et al. (2010) [195]<br>Ikeda et al. (2015) [196]<br>Kim et al. (2013) [198]	Severe sepsis<br>Septic shock	IVIg (5000 mg/day for 3 days) **Total Ig dose:** ~200 mg/kg BW[1]	52<br>410<br>151	- INFM	X			• Fact: All patients were treated with Ig. IL-6, PCT, and CRP were assessed before and after treatment.<br>• CRP, PCT, and IL-6 levels during treatment: Significantly decreased after Ig treatment.

References	Indication	Ig preparation<br>(Dose)<br>Total Ig dose[1]	N	Effect	Significant	Marked change	No difference	Study Results
Reith & Mittelkötter (2001) [202]	Severe sepsis<br>Septic shock	IgM/IgA-enriched Ig (300-400 mL for 3 days)<br>**Total Ig dose:**<br>~600-800 mg/kg BW[1]	67	- PCT<br>- Mortality<br>- HOS stay<br>- ICU stay	 X 	X<br><br>X<br>X		• Fact: Baseline APACHE-II score: 8-29 (median 17) in Ig group vs 8-34 (median 17) in control.<br>• PCT levels: Steadily decreased on days 2-8 to <2 ng/mL in Ig group vs short increases on days 2 and 6 in control.<br>• Mortality: 7/35 (20%) in Ig group (3/18 with severe sepsis and 4/17 with septic shock) vs 16/32 (50%) in control (5/15 and 11/19, respectively) (p <0.05).<br>• Length of HOS stay: 34.6 ± 13.2 days in Ig group vs 39.2 ± 14.4 days in control (NS).<br>• ICU stay: 11.6 ± 6.8 vs 15.6 ± 5.2 days (NS).
Reith et al. (2004) [203]	Abdominal infection with need for surgery	IgM/IgA-enriched Ig (200 mL, 6 h after surgery and 1100 mL over 66 hours continuous infusion)<br>**Total Ig dose:**<br>~866 mg/kg BW[1]	64	- Fever<br>- INFM<br>- PCT<br>- HOS stay		X<br>X<br>X<br>X		• Fact: Baseline APACHE-II score: 9.8 ± 6.8 in Ig group vs 10.4 ± 6.7 in control.<br>• Duration of fever: Reduced in Ig group (34 days) vs control (43 days) (NS)<br>• Duration of HOS: 21 vs 36 days (NS).<br>• TNF-α, leukocytes, thrombocytes, and endotoxin levels decreased faster in Ig group vs control.<br>• IL-8 and IL-10 decreased more in the control group.<br>• PCT levels: Decreased to normal in Ig group but initially decreased and then re-increased during follow-up in control, not reaching normal range.
Tugrul et al. (2002) [206]	Severe sepsis in adults and children ≥10 years	IgM/IgA-enriched Ig (5 mL/kg for 3 days)<br>**Total Ig dose:**<br>750 mg/kg BW	42	- PCT<br>- SOFA<br>- APACHE-II<br>-Mortality G^{neg}<br>-Overall mortality<br>- Septic shock	X 	 <br>X<br>X<br>X<br>X	 X 	• Fact: Baseline APACHE-II and SOFA scores: Similar in Ig group (10.5 ±4.6 and 5.0 ±2.7) vs control (14.0 ±8.5 and 5.7 ±4.0).<br>• PCT level: Decreased significantly in Ig group, but controls showed re-increase (p = 0.001).<br>• SOFA score: 5.0 - 4.0 in Ig group vs 4.5 - 6.0 in controls (NS).<br>• APACHE-II: Decreased in Ig group (15.0 - 8.0) and in control (16.0 to 11.0) (NS).<br>• Mortality in G^{neg} sepsis: 14% in Ig vs 23% in control (p = 0.6).<br>• Overall mortality: 23.8% vs 33.3% (p = 0.7).<br>• Septic shock incidence: 38% vs 57% (p = 0.35).

References	Indication	Ig preparation (Dose) **Total Ig dose[1]**	N	Effect	Significant	Marked change	No difference	Study Results
Toth et al. (2013) [205]	Acute respiratory failure due to septic shock	IgM/IgA-enriched Ig (5 mL/kg for 3 days)<br>**Total Ig dose:**<br>750 mg/kg BW	33	- CRP<br>- PCT<br>- Mortality	X 	 	 X<br>X	• Mortality: High in both groups (71% Ig vs 75% controls).<br>• Patients had multiple organ failures; no change during 8-day observation.<br>• CRP: Decreased significantly faster in Ig group vs controls.<br>• No difference was found for PCT or other parameters.
Ross et al. (1997) [204]	Autoimmune disease	IVIg (400 mg/kg for 3 days)<br>**Total Ig dose:**<br>1200 mg/kg BW	8	- Anti-cytokine	X			• Facts:<br>o Ig contains antibodies against cytokines like IFN-⊚, IL-1a, and IL-6.<br>o Immunomodulatory and ant-inflammatory effects were shown.<br>• Anti-cytokine activity: Significantly increased after Ig treatment (binding activity before and after treatment was IL-1a: 25% to 82%; IFN-α: 16% to 60%; IL-6: 5% to 27%).<br>• Increase positively correlated with Ig dose.<br>• Anti-viral activity: Significantly suppressed by anti-IFN-α antibodies in Ig ($p < 0.02$), relevant for viral infections.
Kress et al. (1999) [199]	Anergic patients undergoing cardiac surgery	IgM/IgA-enriched Ig (start 4 h after surgery: total dose 400 mL via continuous infusion over 53 h)<br>**Total Ig dose:**<br>~267 mg/kg BW[1]	40	- Ig increase<br>- Fever<br>- CRP<br>- Infection incidence	X<br><br><br>X	 X 	 <br>X 	• Ig treatment rapidly reversed postoperative decrease in IgG and IgM, which was significantly different from the control group from days 1 to 7.<br>• Daily maximum body temperature lower in Ig group ($p = 0.067$).<br>• CRP, platelet count, and WBC: NS different between groups.<br>• Overall infection incidence: Significantly lower in Ig group (1/19 [5%]) vs controls (3/21 [15%]), $p = 0.007$.

References	Indication	Ig preparation<br>(Dose)<br>**Total Ig dose**[1]	N	Effect	Significant	Marked change	No difference	Study Results
Willuweit et al. (2019) [209]	Post-transplant patients with vasoplegia	IgM/IgA-enriched Ig (5 mL/kg for 3 days)<br>**Total Ig dose:**<br>750 mg/kg BW	21	- INFM<br>- PCT<br>- Mortality	X<br>X<br>X			• Facts:<br>o In patients with vasoplegia, cardiovascular instability is unresponsive to vasopressors or inotropes.<br>o In patients with septic shock, Igs have anti-inflammatory and anti-endotoxin effects.<br>• Vasopressor need: Significantly decreased after Ig treatment (p <0.001).<br>• IL-6 level: Dropped from 44 ng/mL to 26.1 ng/mL (p = 0.001).<br>• PCT level: Dropped from 63 ng/mL to 20 ng/mL (p <0.001).<br>• 30-day mortality: 14.3%, significantly lower as calculated based on SOFA score (>90%).
Singer et al. (2023) [181]	sCAP patients requiring IMV	IgM/IgA-enriched Ig (3.65 mL/kg for 5 days)<br>**Total Ig dose:**<br>912 mg/kg BW	160	- ANC<br>- ALC<br>- PCT<br>- CRP<br>- Mortality	X<br><br>X<br>X<br>X	 X		• In patients with sCAP and inflammation, Ig significantly faster normalized high ANC (p = 0.016) and PCT (p = 0.027) and stabilized CRP (p = 0.004).<br>• Trend toward faster improvement in high NLR and low ALC vs controls.<br>• In subgroups with inflammation (low ALC, high NLR, high CRP) and low IgM at baseline, Ig treatment significantly decreased mortality and ventilator days vs controls.
Buda et al. (2005) [194]	Sepsis, severe sepsis and septic shock after cardiac surgery	IgM/IgA-enriched Ig (5 mL/kg/d for 3 days)<br>**Total Ig dose:**<br>750 mg/kg BW	66	- APACHE-II<br>- SOFA<br>- INFM<br>-Overall mortality<br>- Pneumonia mortality<br>- Severe sepsis mortality	 <br><br><br><br>X	 <br><br>X<br>X	X<br>X<br>X	• Fact: Patients treated with Ig (n = 22) were matched in APACHE-II score to the controls (n = 44).<br>• Mean APACHE-II score on day 7: from 20.5 to 19.5 vs 21.5 to 20.7 (NS)<br>• Mean SOFA score on day 7: from 11.6 to 10.7 vs 11.7 to 11.1 (NS).<br>• NS difference between groups in inflammatory markers (leukocytes, temperature, cardiac and respiratory rates), the trend of the hemodynamic parameters, or the duration of the ICU stay until day 7.<br>• Overall in mortality: 22.7% in Ig group vs 36.4% in control (NS).<br>• Mortality in patients with severe sepsis: Significantly reduced in Ig group (6.6% vs 37.5%, p = 0.02, n = 47).

References	Indication	Ig preparation (Dose) Total Ig dose[1]	N	Effect	Significant	Marked change	No difference	Study Results
Domizi et al. (2019) [192]	Severe sepsis and septic shock	IgM/IgA-enriched Ig (5 mL/kg/d continuous infusion over 3 days) **Total Ig dose:** 750 mg/kg BW	20	- Microcirculatory dysfunction<br>- IL-6, IL-10<br>- IL-1b, PCT<br>- Other INFM<br>- Mortality<br>- ICU-stay	 <br>X	X<br><br><br>X	 <br><br><br>X<br>X<br>X	• Perfused vessel density for small vessels: Increased after Ig treatment but decreased in placebo group at 72 h (p = 0.140).<br>• Changes in WBC, PCT, and cytokines (IL-1β, TNF-α, IL-8): Similar between groups.<br>• IL-6 and IL-10 levels: Significantly decreased at 72 h only in Ig group<br>• TNF-α: Significantly decreased at 72 h only in the control group.<br>• ICU mortality: Similar between groups, 20% in Ig group vs 22% in placebo (p = 0.999).<br>ICU length of stay: Similar, 19 ± 13 days in Ig group vs 16 ± 12 days in placebo (p = 0.649).
Yavuz et al. (2012) [210]	Sepsis with MODS	IgM/IgA-enriched Ig (5 mL/kg/d over 3 days) **Total Ig dose:** 750 mg/kg BW	118	- APACHE-II<br>- Mortality	X<br>X			• Fact: Mean baseline APACHE-II score was 27.1 ± 2.3 in Ig group (n = 56) vs 27 ± 1.7 in control (n = 62).<br>• APACHE-II score on day 4: Significantly decreased in both groups (16.3 ± 6.3 vs 23.0 ± 7.0).<br>• Overall mortality: Significantly lower in Ig group (42.9% vs 85.5%, p<0.0001).
Werdan et al. (2008) [208] (also previously described in [200,201, 207])	Postcardiac surgery patients with SIRS	IVIg (600 mg/kg on day 1 and 300 mg/kg on day 2 **Total Ig dose**: 900 mg/kg BW	244	- APACHE-II<br>- INFM<br>- Mortality			X<br>X<br>X	• Fact: Patients with APACHE-II >28 were included in this study.<br>• Decrease in the score on day 5 was similar in Ig and control groups.<br>• Mortality: Not significantly different (39.1% in Ig group vs 31.5% in control).<br>• Ig did not attenuate high plasma levels of IL-6, TNF-α, and sTNFR-I/-II.
Schmidt et al. (2021) [15]	Healthy human participants and sCAP patients	IgM/IgA-enriched Ig (HS: 182.6 mg/kg on 5 days) (sCAP: 182.6 mg/kg on 5 days) **Total Ig dose:** 912 mg/kg BW	63	-Complement modulation (healthy participants)<br>-Complement modulation (sCAP patients)		X<br><br>X		• In healthy participants, Ig treatment for 5 consecutive days decreased C3 and C4 serum levels on days 1 and 2.<br>• Decrease was dose-dependent and not observed in controls.<br>• Complement levels slowly returned to baseline during the following 3-9 days.<br>• In 3 patients with sCAP, similar decrease in C3 and C4 levels observed until day 4. Concentrations were back to normal by day 14.

[1] Total dose was calculated assuming a mean body weight value of 75 kg in case total infusion volume was provided, but the mean weight of the patients was missing.

ALC: absolute lymphocyte counts; ANC absolute neutrophil counts; APACHE: acute physiology and chronic health evaluation score; BW: body weight; C5a: complement factor 5a; CRP: C-reactive protein; G^{neg}: Gram-negative bacteria; HOS: hospitalization; ICU: intensive care unit; IFN: interferon; Ig: immunoglobulin; IL: interleukin; IMV: invasive mechanical ventilation; INFM: inflammatory markers; IVIg: intravenous immunoglobulin; MODS: multiple organ dysfunctions; NLR: neutrophil to lymphocyte rate; NS: not significant; PCT: procalcitonine; sCAP: severe community-acquired pneumonia; SIRS: severe systemic inflammatory response syndrome; SOFA: sequential organ failure assessment; sTNFR: soluble tumor necrosis factor receptor; STSS: streptococcal toxic shock syndrome; TNF: tumor necrosis factor; WBC: white blood cell count.
